# Associations of fruit intake with adiposity and cardiometabolic biomarkers in UK Biobank

**DOI:** 10.1186/s12889-024-19505-7

**Published:** 2024-08-16

**Authors:** Eirini Trichia, Fiona MacLean, Aurora Perez-Cornago, Tammy Y. N. Tong, Jonathan R. Emberson, Timothy J. Key, Sarah Lewington, Jennifer L. Carter

**Affiliations:** 1 Clinical Trial Service Unit & Epidemiological Studies Unit, Nuffield Department of Population Health, University of Oxford, Oxford, UK; 2 Cancer Epidemiology Unit, Nuffield Department of Population Health, University of Oxford, Oxford, UK; 3 Health Data Research UK, University of Oxford (HDRUK-Oxford), Oxford, UK

**Keywords:** Fruit, Cardiometabolic, Adiposity, Lipids, Blood pressure, Glycaemia, Inflammation, Oxidative stress, UK Biobank

## Abstract

**Background:**

Fruit consumption has been associated with a lower cardiovascular disease (CVD) risk but the underlying mechanisms are unclear. We investigated the cross-sectional and prospective associations of fruit consumption with markers of adiposity, blood pressure, lipids, low-grade inflammation, glycaemia, and oxidative stress.

**Methods:**

The main analyses included 365 534 middle-aged adults from the UK Biobank at baseline, of whom 11 510, and 38 988 were included in the first and second follow-up respectively, free from CVD and cancer at baseline. Fruit consumption frequency at baseline was assessed using a questionnaire. We assessed the cross-sectional and prospective associations of fruit with adiposity (body mass index, waist circumference and %body fat), systolic and diastolic blood pressure, lipids (low-density and high-density lipoproteins, triglycerides and apolipoprotein B), glycaemia (haemoglobin A1c), low-grade inflammation (C-reactive protein) and oxidative stress (gamma-glutamyl-transferase) using linear regression models adjusted for socioeconomic and lifestyle factors. Analyses were repeated in a subset with two to five complete 24-h dietary assessments (*n* = 26 596) allowing for adjustment for total energy intake.

**Results:**

Fruit consumption at baseline generally showed weak inverse associations with adiposity and biomarkers at baseline. Most of these relationships did not persist through follow-up, except for inverse associations with diastolic blood pressure, C-reactive protein, gamma-glutamyl transferase and adiposity. However, for most mechanisms, mean levels varied by less than 0.1 standard deviations (SD) between high and low fruit consumption (> 3 vs < 1 servings/day) in further adjusted models (while the difference was < 0.2 SD for all of them). For example, waist circumference and diastolic blood pressure were 1 cm and 1 mmHg lower in high compared to low fruit intake at the first follow-up (95% confidence interval: -1.8, -0.1 and -1.8, -0.3, respectively). Analyses in the 24-h dietary assessment subset showed overall similar associations.

**Conclusions:**

We observed very small differences in adiposity and cardiometabolic biomarkers between those who reported high fruit consumption vs low, most of which did not persist over follow-up. Future studies on other mechanisms and detailed assessment of confounding might further elucidate the relevance of fruit to cardiovascular disease.

**Supplementary Information:**

The online version contains supplementary material available at 10.1186/s12889-024-19505-7.

## Background

Cardiovascular disease (CVD) is a leading cause of death and disability worldwide [1], with low fruit intake estimated to be one of the leading causes contributing to about 2 million CVD deaths in 2017 based on approximately 10%-30% higher risk of ischaemic heart disease and stroke in people aged 50–60 years [2]. Therefore, it is important to investigate associations of fruit consumption with major cardiometabolic risk factors to obtain a better understanding of the potential pathways that might link fruit consumption to the risk of CVD.

Conclusive evidence from clinical trials on fruit and cardiometabolic risk factors is limited due to the heterogeneity in the interventions, comparator groups, duration of the trials and small sample sizes. A systematic review of 11 randomised controlled trials showed no effect of an isocaloric increase in fruit consumption on anthropometric markers, while it found evidence that increased fruit consumption might lead to lower weight gain in ad libitum diets [3]. Another systematic review of 36 trials reported small beneficial effects of fruit on markers of inflammation, but with substantial heterogeneity [4]. Evidence from systematic reviews for other markers like blood pressure is more limited to specific fruit e.g. blueberries, for which a systematic review of 11 controlled trials showed small beneficial effects on blood lipids, and diastolic blood pressure (DBP) and no effects on systolic blood pressure (SBP) or glycaemic markers [5]. Several observational studies have also assessed associations between habitual fruit consumption and cardiovascular risk factors, but with limitations too. Overall, the majority of this evidence comes from small or cross-sectional studies that are often missing important covariates such as body mass index (BMI), other dietary factors or total energy intake. For adiposity, three large prospective cohort studies showed inverse associations of fruit intake with body weight gain [6, 7] or the risk of developing obesity [8]. Regarding blood pressure, a meta-analysis of seven prospective cohort studies reported an inverse association between fruit intake and risk of incident hypertension, albeit with significant heterogeneity; stronger associations were reported for follow-up of < 10 years, among men and when hypertension was measured rather than self-reported [9]. Lastly, evidence on associations of fruit intake with blood lipids [10], glycaemia [11, 12], inflammation and oxidative stress [4, 13] is sparse.

We aimed to assess cross-sectional and prospective associations of fruit consumption with adiposity and cardiometabolic biomarkers including blood pressure, lipids, low-grade inflammation, glycaemia and oxidative stress in large subsets of the UK Biobank accounting for important potential confounders to obtain a better understanding of the potential pathways linking fruit consumption to cardiovascular disease.

## Methods

### Study design and population

UK Biobank is a prospective cohort study of initially 502 655 adults, aged 40–69 years, recruited from 2006 to 2010 from 22 assessment centres across the UK through population-based registries (response rate 5.5%) [14]. A subset of over 20 000 adults participated in the resurvey (2012–2013) and a subset of over 70 000 participated in the second follow-up (imaging visit; 2014–2023). Supplemental Figure S1 summarises the data collected in each time point.

### Dietary assessment

Dietary intake was assessed with two types of self-reported assessments: a questionnaire and a validated 24-h dietary assessment (Oxford WebQ). The questionnaire asked about the frequency of consumption of some food groups in the past year and was collected at baseline at the assessment centres. For fruit intake, participants were asked how many pieces of fresh fruit they would eat on average per day in the past year and were given examples of how to count a piece [15]. For this analysis, it was assumed that a piece would equal a serving. The 24-h dietary assessment asked more detailed questions about foods and beverages consumed in the previous 24 h and was repeated to a maximum of five times between April 2009 and June 2012. The questionnaire was administered in the whole cohort, so it was used in the main analyses, while the 24-h assessment was administered in a subset and is included in secondary analyses. More information on the 24-h assessment can be found in the Supplemental Methods.

### Adiposity and cardiometabolic biomarkers

Available markers of adiposity, lipidaemia, inflammation, oxidative stress, glycaemia and blood pressure in UK Biobank were selected as outcomes for this analysis when deemed informative for predicting the risk of CVD and without substantial overlap. Anthropometric measures were available at baseline and both follow-ups. BMI was derived from body weight (kg) divided by the square of standing height (m^2^). Waist circumference (WC) was measured at the narrowest part of the trunk, or the umbilicus if the former could not be identified, using a Seca 200 cm tape measure. The percentage of body fat was estimated using the bio-impedance method with a Tanita BC418MA analyser. Blood pressure (mmHg) was measured twice either automatically (Omron 705 IT, OMRON Healthcare Europe) or manually (when automatic readings were not available) and the average of the two measurements was used.

Serum biomarkers were related to lipids, low-grade inflammation (C-reactive protein – CRP) and oxidative stress (gamma-glutamyl transferase—GGT) and were measured at baseline and first follow-up. Blood samples were collected at the assessment centres and refrigerated until transferred to the central laboratory at Stockport. Apolipoprotein B (ApoB) and CRP were measured with an immune-turbidimetric method and other lipids [low-density lipoprotein cholesterol (LDL-C), high-density lipoprotein cholesterol (HDL-C) and triglycerides] and GGT with an enzymatic method (Beckman Coulter AU5800 analyser). Haemoglobin A1c (HbA1c) was measured with high-performance liquid chromatography (Bio-Rad Variant II Turbo analyser) using packed red blood cell samples.

### Statistical analysis

Baseline characteristics of the participants are presented as age- and sex-standardised means (SD) or frequencies (%) and their associations with baseline fruit consumption were assessed with age- and sex-adjusted linear regression models.

In the main analysis using the frequency questionnaire, fruit intake was treated as a four-category variable (< 1, 1, 2, ≥ 3 servings/day). Outcomes were treated as continuous variables. Right-skewed outcomes (i.e. triglycerides, CRP, and GGT) were log-transformed and their effect sizes and 95% confidence intervals (CIs) were back-transformed with exponentiation. Four sets of covariates were used in linear regression models of the association of fruit consumption with the cardiometabolic outcomes: 1) adjusted for age and sex; 2) additionally adjusted for ethnicity, quintiles of the Townsend deprivation index, educational level (four levels), smoking status (never, previous, current), alcohol consumption (five categories), and physical activity (low, moderate, high); 3) additionally adjusted for categories of intakes of vegetables, non-oily fish, oily fish, red unprocessed meat, processed meat, cheese, whole grains, refined grains, tea, coffee, type of fat spread used, and use of dietary supplements; 4) additionally adjusted for BMI (continuous) when the outcome was not BMI. Adjustment for BMI and physical activity can act as a proxy for total energy intake, which is not available from the questionnaire (as it does not include a comprehensive assessment of the diet). Missing values of categorical variables were included as a separate category and those for continuous variables were excluded. To examine the shapes of the associations, the adjusted means of the outcomes were plotted (with y-axes standardised to a range of 1 SD) against levels of fruit intake and we assessed the difference between the highest and the lowest category of fruit consumption and whether there was a linear trend across all categories. To compare the strength of the associations across cardiometabolic outcomes, adjusted mean differences between low and high fruit intake were also expressed as a change in SD, calculated as the adjusted mean difference divided by the SD of the outcome. P values were corrected for multiple testing using the Benjamini–Hochberg method to control the false discovery rate [16].

In sensitivity analyses, for each of the four models described above, fruit consumption and dietary covariates (including total energy intake) were derived from the mean of two to five 24-h dietary assessments (rather than the frequency questionnaire). More information on this analysis is provided in the Supplemental Methods. Secondary analyses included comparison of models with and without total energy intake and excluding any records that reported untypical diet the previous day. The Spearman rank correlation coefficient was used to compare the agreement in the rankings of participants on their fruit consumption between the frequency questionnaire and the 24-h dietary assessment tool. Further sensitivity analyses of the cross-sectional associations included: repeating the adiposity and blood pressure analyses limited to those who took part in the second follow-up; excluding participants with self-reported diabetes or insulin at baseline; repeating analyses of blood pressure and lipids excluding, respectively, those taking antihypertensive or lipid-lowering drugs at baseline; and repeating analyses of CRP excluding those with baseline CRP > 10 mg/l (to exclude the possibility of an acute infection) [17].

## Results

**Fig. 1:**
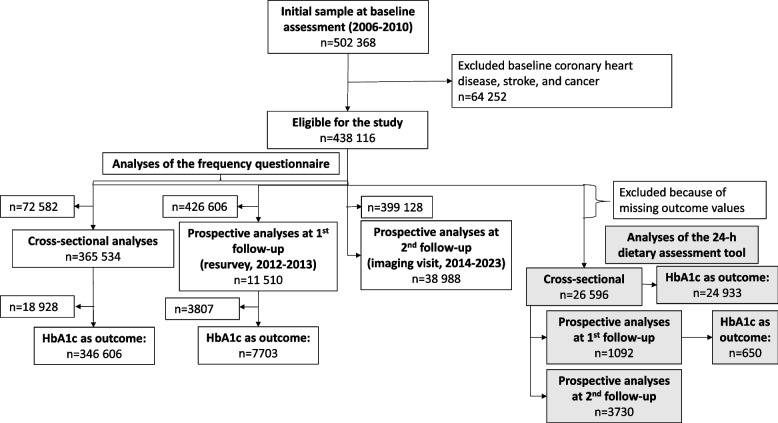
Participant flowchart. *HbA1c *haemoglobin A1c

After exclusion of participants with self-reported CVD or cancer at baseline and those with missing values for the cardiometabolic biomarkers at each time point, the main analysis included 365 534 participants at baseline, 11 510 at first follow-up, and 38 988 at second follow-up (Fig. 1). The mean (SD) of fruit consumption at baseline was 2.2 (1.6) servings/day. Compared with participants consuming < 1 fruit serving/day, participants who reported consuming ≥ 3 fruit servings/day were more likely to be women, more educated, less deprived and less likely to be current smokers or high alcohol consumers (age- and sex-adjusted) (Table 1). They also reported consuming more vegetables, whole grains, fish, and dietary supplements, and less refined grains, meat and regular coffee. The subset of participants answering the 24-h dietary assessment were more educated, less likely to be current smokers and had slightly lower WC, body fat percentage, SBP, CRP and GGT than the main analysis population but the distribution of baseline characteristics across categories of fruit intake was similar to that of the main population (Supplemental Tables S1 & S2**)**. There was moderate agreement in the ranking of the participants’ fruit consumption between the frequency questionnaire and the 24-h dietary assessment (Spearman correlation: 0.62). The mean levels of the cardiometabolic biomarkers were broadly similar across the different time points (Supplemental Table S2). On average SBP and HbA1c slightly increased and DBP, slightly decreased over time.

**Table 1:** Baseline characteristics in the total sample (*n* = 365 534) and by extreme categories of fruit intake

	**Fruit intake (n participants)**		**All participants** **(365 534)**	**P**^**a**^
	** < 1 serv/d** **(33 793)**	**≥ 3 serv/d** **(130 865)**		
Age, years	54.1 (8.2)	56.8 (7.8)	56.0 (8.1)	< 0.001
Women	13 371 (40%)	81 327 (62%)	199 361 (55%)	< 0.001
White ethnicity	32 013 (95%)	122 464 (94%)	344 078 (95%)	< 0.001
Higher qualification	17 943 (53%)	81 161 (62%)	222 005 (61%)	< 0.001
Highest quintile (Q5) of deprivation index	8943 (26%)	24 634 (19%)	71 777 (20%)	< 0.001
Current smokers	8395 (25%)	8484 (6%)	37 799 (10%)	< 0.001
High weekly alcohol consumption (> 14 units/wk)	15 563 (46%)	37 866 (29%)	127 449 (35%)	< 0.001
< 10 MET-hours/wk	8511 (25%)	20 126 (15%)	68 911 (19%)	0.227
Dietary factors from the frequency questionnaire				
Vegetables ≥ 3 servings/d	5206 (15%)	55 388 (42%)	113 036 (31%)	< 0.001
Whole grains ≥ 3 servings/d	2851 (8%)	21 788 (17%)	54 501 (15%)	< 0.001
Refined grains ≥ 3 servings/d	4545 (13%)	6855 (5%)	28 532 (8%)	< 0.001
Cheese ≥ 5 times/wk	4500 (13%)	16 370 (13%)	48 103 (13%)	< 0.001
Non-oily fish ≥ 1 time/wk	3962 (12%)	25 679 (20%)	58 952 (16%)	< 0.001
Oily fish ≥ 1 time/wk	3244 (10%)	30 971 (24%)	63 499 (17%)	< 0.001
Red unprocessed meat ≥ 1 time/wk	27 781 (82%)	102 208 (78%)	295 827 (81%)	< 0.001
Processed meat ≥ 1 time/wk	15 023 (44%)	31 321 (24%)	113 039 (31%)	0.024
Type of spread consumed: Butter	14 747 (44%)	42 940 (33%)	134 185 (37%)	0.357
Caffeinated coffee ≥ 4 cups/d	7582 (22%)	18 739 (14%)	57 811 (16%)	< 0.001
Decaffeinated coffee ≥ 3 cups/d	1761 (5%)	8682 (7%)	22 151 (6%)	0.111
Tea ≥ 5 cups/d	10 640 (31%)	39 039 (30%)	108 394 (30%)	0.575
Dietary supplements	12 526 (37%)	74 867 (57%)	185 696 (51%)	< 0.001
Anti-hypertensive medication	5577 (17%)	24 444 (19%)	64 267 (18%)	< 0.001
Lipid-lowering medication	4155 (12%)	18 391 (14%)	48 362 (13%)	< 0.001
Diabetes	1136 (3%)	6545 (5%)	15 414 (4%)	< 0.001

*MET *metabolic equivalents of task

^a^Age- and sex-adjusted linear regression models of covariates on fruit consumption

Means (SD) for continuous variables and frequencies (%) for categorical variables have been adjusted for age (5 years groups) and sex. Age and sex estimates are not adjusted for age and sex respectively

All variables have < 5% missing values apart from MET-hours/week with 22% missing values

**Fig. 2:**
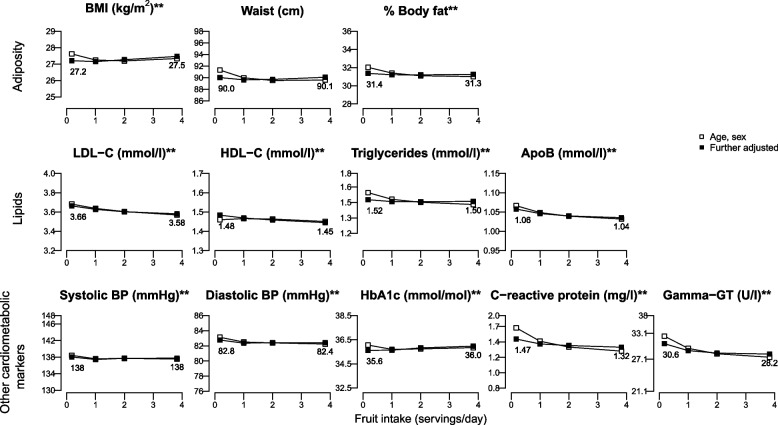
Adjusted means of adiposity and cardiometabolic biomarkers at BASELINE (2006–2010) by BASELINE fruit intake (servings/day) assesed from a frequency questionnaire. ApoB, apolipoprotein B; BMI, body mass index; BP, blood pressure; FDR, false discovery rate; GT, glutamine transferase; HbA1c, haemoglobin A1c; HDL-C, high-density lipoprotein cholesterol; LDL-C, low-density lipoprotein cholesterol. *n* = 365 534 for all the outcomes apart from HbA1c with *n* = 346 606; *FDR-adjusted *P* < 0.05 for the age, sex adjusted model; **FDR-adjusted *P* < 0.05 for both models. Further-adjusted model was additionally adjusted for ethnicity, quintiles of the Townsend deprivation index, educational level, smoking status, alcohol consumption, physical activity, vegetable intake, type of fat spread used, intakes of non-oily fish, oily fish, processed meat, cheese, whole grain, and refined grains, tea, coffee, and dietary supplements. Estimates for the log-transformed triglycerides, C-reactive protein, and gamma-GT were back-transformed with exponentiation. In each panel the y-axis extends to the population mean ± x standard deviations

**Fig. 3:**
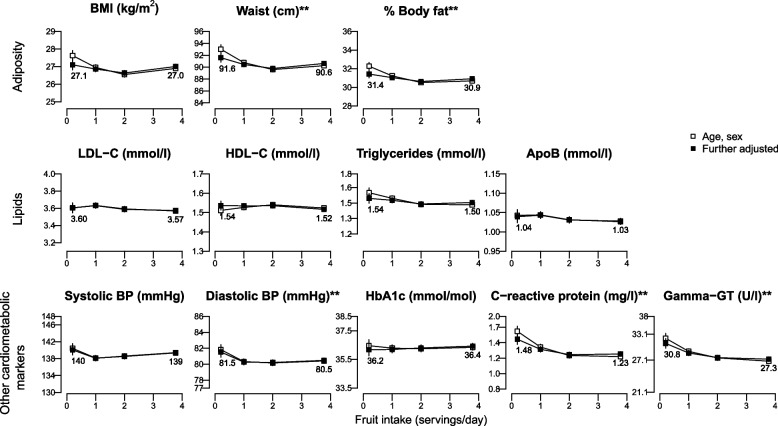
Adjusted means of adiposity and cardiometabolic biomarkers at FIRST FOLLOW-UP (2012–2013) by BASELINE fruit intake (servings/day) assessed from a frequency questionnaire. ApoB, apolipoprotein B; BMI, body mass index; BP, blood pressure; FDR, false discovery rate; GT, glutamine transferase; HbA1c, haemoglobin A1c; HDL-C, high-density lipoprotein cholesterol; LDL-C, low-density lipoprotein cholesterol. *n* = 11 510 for all the outcomes apart from HbA1c with *n* = 7703; *FDR-adjusted *P* < 0.05 for the age, sex adjusted model; **FDR-adjusted *P* < 0.05 for both models. Further-adjusted model was additionally adjusted for ethnicity, quintiles of the Townsend deprivation index, educational level, smoking status, alcohol consumption, physical activity, vegetable intake, type of fat spread used, intakes of non-oily fish, oily fish, processed meat, cheese, whole grain, and refined grains, tea, coffee, and dietary supplements. Estimates for the log-transformed triglycerides, C-reactive protein, and gamma-GT were back-transformed with exponentiation. In each panel the y-axis extends to the population mean ± x standard deviations

**Fig. 4:**
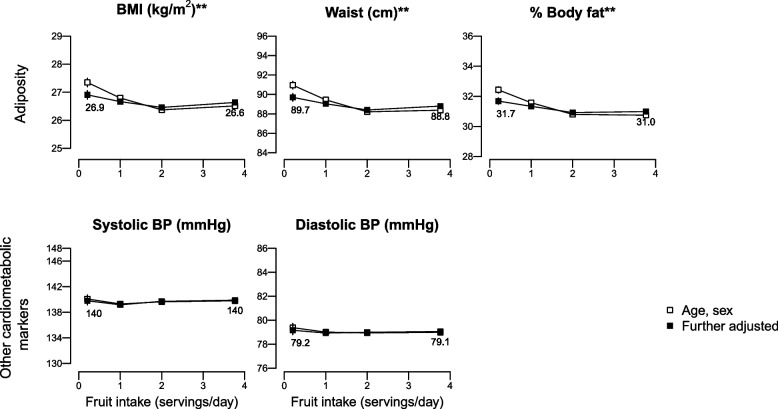
Adjusted means of adiposity and cardiometabolic biomarkers at SECOND FOLLOW-UP (2014–2023) by BASELINE fruit intake (servings/day) assessed from a frequency questionnaire. BMI, body mass index; BP, blood pressure; FDR, false discovery rate; *n* = 38 988; *FDR-adjusted *P* < 0.05 for the age, sex adjusted model; **FDR-adjusted *P* < 0.05 for both models. Further-adjusted model was additionally adjusted for ethnicity, quintiles of the Townsend deprivation index, educational level, smoking status, alcohol consumption, physical activity, vegetable intake, type of fat spread used, intakes of non-oily fish, oily fish, processed meat, cheese, whole grain, and refined grains, tea, coffee, and dietary supplements. In each panel the y-axis extends to the population mean ± x standard deviations

The only dose–response associations we observed were with baseline CRP and GGT, which showed evidence of linear trends (Supplemental Tables S3-S5); there were no dose–response associations with any other biomarkers. When comparing high with low intakes of fruit, overall to see if there were any differences when the relationship was non-linear, in fully adjusted analyses we observed weak inverse associations between higher fruit consumption and cardiometabolic risk markers, which varied by less than 0.1 SD for most of the markers apart from GGT (baseline and first follow-up) and CRP (first follow-up), for which they varied by a range of 0.13–0.18 SDs (Fig. 2; Supplemental Tables S3 and S6). For example, WC and DBP were 0.97 cm (95% CI: -1.82, -0.13) and 1.05 mmHg (-1.78, -0.32) lower in high vs low fruit consumers after 4y of follow-up (Fig. 3, Supplemental Table S4**)**. As an exception, in multi-adjusted models high baseline fruit consumption (≥ 3 servings/day) was associated with slightly higher baseline BMI and HbA1c compared with low fruit consumption (< 1 serving/day) (mean difference [95% CI]: 0.26 kg/m^2^ [0.20, 0.32] and 0.34 mmol/mol [0.26, 0.43], Fig. 2, Supplemental Table S3). Of note, while adjustments for sociodemographic and lifestyle factors in addition to age and sex further attenuated the associations for most biomarkers, when the outcome was baseline BMI or HbA1c, further adjustment reversed the associations. Fully adjusted prospective associations showed a similar positive association for HbA1c after 4y of follow-up, but with lower precision (Fig. 3, Supplemental Table S4) and an inverse association with BMI at 10y of follow-up (-0.27 kg/m^2^ [-0.45, -0.10] Fig. 4, Supplemental Table S5). Inverse associations with blood lipids and SBP at baseline were not retained in prospective analyses. Further adjustment for BMI did not materially change the observed associations, apart from the cross-sectional associations with WC and %body fat, which became stronger (Supplemental Table S3).

Results from most of the sensitivity analyses were not materially different from the main analysis (Supplemental Table S7). When the cross-sectional analyses were performed in the subset of the second follow-up, fruit intake was no longer associated with baseline BMI, while in the subset without baseline self-reported diabetes, a weak inverse association was observed between high fruit intake and WC (-0.16 cm [-0.31, -0.01]), and the association with HbA1c was now inverse (-0.09 mmol/mol [-0.15, -0.03]; Supplemental Table S7).

Compared to the main analyses, analyses in the 24-h assessment subset did not show any associations of fruit with BMI at any time point or with WC, CRP and GGT at follow-up, but more cardiometabolic markers displayed linear trends across quintiles of fruit intake including lower WC (baseline), % body fat (baseline and 10y), LDL-C (baseline), triglycerides (baseline and 4y), and ApoB (baseline) (Supplemental Tables S8-S10 and Supplemental Figures S2-S4). Associations with SBP and DBP after 4 years of follow-up were positive when using the 24-h dietary assessment as opposed to those of the main analyses. Adjusting for total energy intake (Supplemental Table S11) or excluding records reporting untypical diet (Supplemental Figures S5-S7) did not materially change the results.

## Discussion

### Summary of results

In this study, mean differences in adiposity and cardiometabolic biomarkers between those reporting high fruit consumption vs low were very small and most of them did not persist in prospective analyses. For most of the cardiometabolic risk markers, mean levels varied by less than 0.1 SD between high and low fruit consumption once differences in socio-demographic factors and other dietary factors were taken into account (while the difference was < 0.2 SD for all of them). For most of the biomarkers, high fruit consumers had slightly lower mean levels than low fruit consumers, but for baseline BMI and HbA1c the opposite was observed. There was evidence of inverse linear associations only between fruit consumption and CRP and GGT.

### Interpretation in the context of previous evidence

In this study, baseline high fruit intake was associated with a slightly higher baseline BMI by 0.26 kg/m^2^ and a lower BMI at 10 years by 0.27 kg/m^2^. For WC and % body fat we reported weak inverse associations consistently across time points after adjusting for BMI. The China Kadoorie Biobank of approximately 450 000 Chinese adults reported a 0.5 kg/m^2^ higher BMI and 0.9 cm higher WC (not adjusted for BMI) at baseline among those who reported consuming fruit daily compared to those who never consumed fruit [18], while a Canadian study of about 26 000 adults reported a 0.12 kg/m^2^ lower BMI and 0.4 cm lower WC at baseline with higher reported fruit consumption [19]. In a joint analysis of three American cohorts of about 130 000 adults, higher fruit consumption was inversely associated with weight gain over 4 years [6], while in another American cohort of women, higher fruit consumption was associated with lower risk of developing overweight or obesity after 16 years [8]. Finally, a study across 10 European countries did not find an association between fruit and body weight [7]. It has been previously shown that healthy foods such as fruit and vegetables might be over-reported due to social desirability bias [20]. If this bias differs by BMI status, it might result in reverse causality in a cross-sectional analysis if individuals with overweight/obesity over-report fruit intake more than others. Associations of fruit consumption with lipid levels have not been broadly studied in large observational studies. In the Korean Genome and Epidemiology Study (KoGES), high fruit intake was associated with lower risk of hypertriglyceridaemia over 8 years, but not with HDL-C levels [10] while in this study we did not observe any clinically meaningful associations. While the China Kadoorie Biobank reported a 3.2 mm Hg lower SBP [18] for daily vs no fruit consumption, a cross-sectional analysis among about 300 000 European adults from the European Prospective Investigation into Cancer (EPIC) found 0.6 mm Hg lower SBP for the fifth quintile of fruit intake compared to the first [21]. The reason why the associations that we (0.45 mm Hg lower SBP in multi-adjusted models) and EPIC reported are much smaller than the one observed among Chinese adults is not clear, but it might be related to different confounding patterns in different populations, or with the fact that the association among the Chinese adults was further adjusted for WC. Contrary to our main analysis and previous evidence, high fruit intake was associated with a higher SBP and DBP at 4 years in the 24-h dietary assessment subset; the reasons for that are not clear and it might be due to chance due to the large number of tests performed.

A potential explanation of an inverse association of fruits with certain cardiometabolic biomarkers is their fibre content. Clinical trials have shown that total dietary fibre exerts beneficial effects on multiple cardiometabolic factors including lowering body weight, SBP and total and LDL cholesterol [22]. Suggested pathways include reduced or slower absorption of lipids and sugars, higher satiety [23], but also effects on the gut microbiome [24], which has been associated with cardiometabolic health [25]. Future research on these suggested pathways could elucidate more on their relevance to the association of fruit consumption with cardiometabolic biomarkers.

### Clinical significance

After accounting for multiple testing, we identified several associations of fruit consumption with cardiometabolic risk markers, but we have noted that these associations are mostly of small magnitude with uncertain clinical significance. For example, high fruit intake was associated with a 0.27 kg/m^2^ lower BMI after 10 years of follow-up. Considering that BMI has been previously associated with a 56% (HR = 1.56; 95% CI: 1.54, 1.58) higher 14-year risk of cardiovascular mortality per 5 kg/m^2^ above 25 kg/m^2^ among non-smoking Europeans [26], it can be assumed that the approximate decrease in cardiovascular mortality when BMI decreases by 0.27 kg/m^2^ would be, at most, about 2.5%. Similarly, the epidemiological differences in CVD mortality risk associated with the differences seen in cardiometabolic biomarkers between high and low fruit consumers would be 2% for a 1 cm decrease in WC over 10 years [27], 4% for 1 mmHg lower DBP over 4 years [28] or 5% for 3.5 U/l lower GGT over 10 years (assuming GGT is causally related to CVD) [29]. These risk reductions are small and it is uncertain whether, in combination, they might explain the moderate increase in the risk of ischaemic heart disease and stroke (approximately 10–30%, respectively, among people 50–60 years) attributed to a diet low in fruits observed in the Global Burden of Disease Study 2017 [2]. However, it is important to note that the reported associations from previous studies might be inflated if they did not adequately account for confounding.

### Strengths and limitations

In this study we were able to assess associations of fruit intake with adiposity and cardiometabolic biomarkers with high precision through large sample sizes among approximately 400 000, 10 000 and 40 000 participants at baseline, first and second follow-up respectively, but also with high depth in subsets of 26 500, 1 000 and 3 500 respectively with a detailed assessment of fruit intake. We also sought to minimise confounding by adjusting for important potential confounders including multiple dietary factors (including energy intake in secondary analyses), and BMI, which might partly account for any social desirability bias and which many previous studies missed.

Limitations of this study include the lack of detail in the primary dietary assessment tool we used (i.e. the questionnaire), which only assessed frequency of total fruit intake. It might be of interest to investigate associations of types of fruit, as certain types might exert more beneficial effects on cardiovascular risk than others. However, it is also important to consider potential challenges of studying associations of types of fruit including lower intakes and different patterns of confounding, which would make comparison of results across different types more difficult. Another limitation is that the subsets of the two follow-ups were not randomly selected from the baseline cohort, which makes comparison of the results more challenging. Nevertheless, outcome values did not materially differ across time points and sensitivity analyses of the cross-sectional associations in the follow-up subset showed overall similar associations with that in the baseline cohort. The two dietary assessment tools were not directly comparable, as the one included general frequency questions over the past months and the 24-h assessment included a more detailed assessment of the dietary intake over the past 24 h and they assessed quantities consumed differently (the questionnaire included a more crude assessment of pieces of total fruit consumed). Despite these differences, the ranking of the participants based on their fruit consumption was deemed sufficiently similar and was consistent with previous reports of agreement between different methods of dietary assessment [30]. Lastly, even though we adjusted for various potential confounders, residual confounding is still likely (e.g. from the imperfect measurement of dietary intake and physical activity with the self-reported methods used).

## Conclusion

We reported associations of small magnitude between fruit intake and markers of adiposity, blood pressure, inflammation and oxidative stress, which are not large enough to explain the size of reported associations of a diet low in fruit with cardiometabolic disease. Our results do not support any substantial impact of fruit on the risk of CVD through the studied risk factors in this study population. More research in different populations and on more potential cardiometabolic risk factors, as well as considering specific fruit groups is warranted to further elucidate any underlying pathways linking fruit consumption to CVD.

### Supplementary Information

Supplementary Material 1.

## Data Availability

All results from this analysis are returned to UK Biobank within 6 months of publication, at which point they can be made available to other researchers upon reasonable request. UK Biobank is an open access resource, and researchers can apply to use the dataset at http://ukbiobank.ac.uk/register-apply/.

## Abbreviations

ApoB: Apolipoprotein B
BMI: Body mass index
CI: Confidence interval
CRP: C-reactive protein
CVD: Cardiovascular disease
DBP: Diastolic blood pressure
GGT: Gamma-glutamyl transferase
HbA1c: Haemoglobin A1c
HDL-C: High-density lipoprotein cholesterol
LDL-C: Low-density lipoprotein cholesterol
SBP: Systolic blood pressure
WC: Waist circumference
