# Supplementary material for: Associations of fruit intake with adiposity and cardiometabolic biomarkers in UK Biobank
Source: BMC Public Health. 2024 Aug 16;24:2227. doi: 10.1186/s12889-024-19505-7 (PMC11328357; doi:10.1186/s12889-024-19505-7)
Supplement: Supplementary file 1 — Supplementary Material 1. [file 12889_2024_19505_MOESM1_ESM.pdf]

Associations of fruit intake with adiposity and cardiometabolic biomarkers in UK Biobank

Eirini Trichia, Fiona MacLean, Aurora Perez-Cornago, Tammy YN Tong, Jonathan R Emberson, Timothy J Key, Sarah Lewington, Jennifer L Carter

Supplemental Material

Table of Contents

	Page
**Supplemental Methods**	3
**Supplemental Table S1**. Baseline characteristics of 26 596 participants with 2 to 5 24-hour dietary assessments and by extremes of fruit intake	4
**Supplemental Table S2**. Descriptive statistics of cardiometabolic risk factors at different time points in the subsets with dietary data from the frequency questionnaire and the 24-hour dietary assessment	5
**Supplemental Table S3**. Associations of BASELINE fruit consumption with cardiometabolic risk factors at BASELINE (2006-2010) in the subset with dietary data from the frequency questionnaire	6
**Supplemental Table S4**. Associations of BASELINE fruit consumption with cardiometabolic risk factors at FIRST FOLLOW-UP (2012-2013) in the subset with dietary data from the frequency questionnaire	8
**Supplemental Table S5**. Associations of BASELINE fruit consumption with cardiometabolic risk factors at SECOND FOLLOW-UP (2014-2023) in the subset with dietary data from the frequency questionnaire	10
**Supplemental Table S6.** Fully adjusted mean differences in cardiometabolic risk factors at different time points in the subsets with dietary data from the frequency questionnaire, presented as changes in SD	11
**Supplemental Table S7**. Sensitivity analyses for associations of BASELINE fruit consumption with cardiometabolic risk factors at baseline (2006-2010) in the subset with dietary data from the frequency questionnaire	12
**Supplemental Table S8**. Cross-sectional associations of BASELINE fruit consumption with cardiometabolic risk factors at BASELINE (2009-2010) in the subset with dietary data from 2 to 5 WebQ 24-hour dietary assessments (2009-2012)	15
**Supplemental Table S9**. Prospective associations of BASELINE fruit consumption with cardiometabolic risk factors at FIRST FOLLOW-UP (2012-2013) in the subset with dietary data from 2 to 5 WebQ 24-hour dietary assessments (2009-2012)	17
**Supplemental Table S10**. Prospective association of BASELINE fruit consumption with cardiometabolic risk factors at SECOND FOLLOW-UP (2014-2023) in the subset with dietary data from 2 to 5 WebQ 24-hour dietary assessments (2009-2012)	19
**Supplemental Table S11**. Summary of associations between fruit intake at baseline and cardiometabolic risk factors at all time-points, by method of fruit intake measurement, comparing ~3 vs <1 servings per day	20
**Supplemental Figure S1**. Timeline of data collection in UK Biobank	21
**Supplemental Figure S2.** Adjusted means of cardiometabolic risk factors at BASELINE (2009−2010) by BASELINE mean fruit intake (g/day) among those completing 2 to 5 WebQ 24−h dietary assessments (2009−2012)	22

Supplemental Figure S3. Adjusted means of cardiometabolic risk factors at FIRST FOLLOW−UP (2012−2013) by BASELEINE mean fruit intake (g/day) among those completing 2 to 5 WebQ 24−hdietary assessments (2009−2012) 23
Supplemental Figure S4. Adjusted means of cardiometabolic risk factors at SECOND FOLLOW−UP (2014−2023) by BASELINE mean fruit intake (g/day) among those completing 2 to 5 WebQ 24−hdietary assessments (2009−2012) 24
Supplemental Figure S5. Adjusted means of cardiometabolic risk factors at BASELINE (2009−2010) by BASELINE mean fruit intake (servings/day) among those completing 2 to 5 WebQ 24−h dietary assessments (2009−2012), with and without restriction for assessments reporting typical diet only 25
Supplemental Figure S6. Adjusted means of cardiometabolic risk factors at FIRST FOLLOW−UP (2012−2013) by BASELEINE mean fruit intake (servings/day) among those completing 2 to 5 WebQ 24−h dietary assessments (2009−2012), with and without restriction for assessments reporting typical diet only 26
Supplemental Figure S7. Adjusted means of cardiometabolic risk factors at SECOND FOLLOW−UP (2014−2023) by BASELINE mean fruit intake (servings/day) among those completing 2 to 5 WebQ 24−h dietary assessments (2009−2012), with and without restriction for assessments reporting typical diet only 27

Supplemental Methods

Description of the 24-hour dietary assessment

The 24-hour dietary assessment tool asked about consumption of 206 foods and 32 beverages in the previous 24 hours. This included 19 fresh, frozen, canned, dried or cooked fruits[1], recorded as whole fruits where possible and servings if not e.g. fruit salad. It was added to the assessment centre protocol from April 2009 and also emailed to all participants with a valid email address four times between February 2011 and June 2012 (response rate for each cycle: 26-33%)[2]. A serving of fruit was assumed to be 80g as per the Food Standards Agency definition[3].

Statistical analyses with the 24-hour dietary assessment

In the analyses that used the 24-hour dietary assessment, only individual assessments with total energy intakes <20 000 KJ for men and <18 000 KJ for women were included. Participants who attended the assessment centre before this tool was introduced, or did not have at least two dietary assessments inside the estimated energy intake range were excluded. Food servings were converted to grams as previously done[4] and mean daily intakes of foods were calculated from the eligible dietary assessments. Each outcome at baseline (n=26 596) and the two follow-ups (n=1902 and 3730 respectively) was regressed on fifths of fruit intake. Fruit servings rather than grams per day, are presented in tables and figures to aid comparison with the main analysis.

References

1. Greenwood DC, Hardie LJ, Frost GS, et al. Validation of the Oxford WebQ Online 24-Hour Dietary Questionnaire Using Biomarkers. *American Journal of Epidemiology* 2019; **188**(10): 1858-67.
2. Carter JL, Lewington S, Piernas C, et al. Reproducibility of dietary intakes of macronutrients, specific food groups, and dietary patterns in 211 050 adults in the UK Biobank study. *J Nutr Sci* 2019; **8**: e34.
3. Food Standards Agency, 2020, The Eatwell Guide, . https://www.food.gov.uk/sites/default/files/media/document/eatwell-guide-master-digital.pdf.
4. Perez-Cornago A, Pollard Z, Young H, et al. Description of the updated nutrition calculation of the Oxford WebQ questionnaire and comparison with the previous version among 207,144 participants in UK Biobank. *European journal of nutrition* 2021; **60**(7): 4019-30.

Supplemental Table S1. Baseline characteristics of 26 596 participants with 2 to 5 24-hour dietary assessments and by extremes of fruit intake.

	Fruit intake quintile (n participants) Q1 (5320)	Q5 (5309)	All participants (26 596)	P value [a]
Fruit servings/day, median (range)	0.4 (0-0.8)	4.4 (3.5 - 19.6)	2.0 (0-19.6)	
Age, years	53.6 (8.1)	56.9 (7.6)	55.7 (8.0)	<0.001
Female	2,600 (49%)	3,098 (58%)	15,002 (56%)	<0.001
White ethnicity	5,095 (96%)	5,028 (95%)	25,382 (95%)	<0.001
Higher qualification	3,662 (69%)	3,987 (75%)	19,542 (73%)	<0.001
Highest quintile (Q5) of deprivation index	998 (19%)	937 (18%)	4,497 (17%)	<0.001
Current smokers	607 (11%)	249 (5%)	1,796 (7%)	<0.001
Alcohol consumption g/day	21 (23)	13 (16)	16 (19)	<0.001
<10 MET-hours/week	2,262 (43%)	1,619 (30%)	9,736 (37%)	<0.001
Dietary factors				
Servings per day (grams per serving)				
Vegetables (80 g)	1.9 (1.4)	3.3 (2.1)	2.5 (1.7)	<0.001
Whole grains (36 g)	1.3 (1.3)	2.0 (1.6)	1.8 (1.5)	<0.001
Refined grains (36 g)	2.3 (1.9)	1.8 (1.8)	2.0 (1.9)	<0.001
Servings per week (grams per serving)				
Cheese (30 g)	4.0 (4.0)	4.4 (4.4)	4.2 (4.0)	<0.001
Non-oily fish (140 g)	1.1 (1.6)	1.1 (1.6)	1.1 (1.5)	0.018
Oily fish (140 g)	0.5 (0.9)	0.8 (1.2)	0.6 (1.1)	<0.001
Red meat (70 g)	4.2 (4.3)	3.2 (3.7)	3.6 (3.69)	<0.001
Processed meat (70 g)	1.9 (2.5)	1.4 (2.0)	1.6 (2.2)	<0.001
Caffeinated coffee cups/day	1.4 (1.4)	1.4 (1.3)	1.4 (1.3)	0.019
Decaffeinated coffee cups/day	0.3 (0.7)	0.3 (0.8)	0.3 (0.8)	<0.001
Tea cups/day	2.2 (1.8)	2.4 (1.8)	2.3 (1.7)	0.008
Dietary supplements	2,149 (40%)	2,727 (51%)	12,359 (46%)	<0.001
Anti-hypertensive medication	958 (18%)	809 (15%)	4,184 (16%)	<0.001
Lipid-lowering medication	760 (14%)	677 (13%)	3,395 (13%)	<0.001
Diabetes	206 (4%)	220 (4%)	931 (4%)	<0.001

MET-hours; Metabolic Equivalent Task hours
Values are standardized to the age and sex distribution of the main study population (after exclusions for baseline disease and missing outcomes). Age and sex estimates are not adjusted for age and sex respectively.
Continuous variables are presented as standardized mean (sd).
All variables have <0.5% missing values apart from MET-hours/week with <5% missing values
[a] Age- and sex-adjusted linear regression models of covariates on fruit consumption. Age and sex variables are not adjusted for age and sex respectively.

Supplemental Table S2. Descriptive statistics of cardiometabolic risk factors at different time points in the subsets with dietary data from the frequency questionnaire and the 24-hour dietary assessment

	Baseline (2006-2010)		1st follow-up (2012-2013)		2nd follow-up (2014-2023)	
	Frequency questionnaire<br>n=365 534	24-h dietary assessment<br>n=26 596	Frequency questionnaire<br>n=11 510	24-h dietary assessment<br>n=1092	Frequency questionnaire<br>n=38 988	24-h dietary assessment<br>n=3730
Markers of adiposity						
Body mass index (kg/m²)	27.3 (4.7)	26.7 (4.6)	26.9 (4.4)	26.4 (4.1)	26.6 (4.4)	26.3 (4.3)
Waist circumference (cm)	89.9 (13.3)	88.2 (13.3)	90.4 (12.9)	89.6 (12.5)	88.8 (12.7)	87.3 (12.6)
Body fat (%)	31.2 (8.5)	30.8 (8.4)	30.9 (8.3)	30.3 (8.1)	31.1 (8.2)	30.6 (8.1)
Lipids						
LDL-C (mmol/l)	3.6 (0.8)	3.6 (0.8)	3.6 (0.9)	3.6 (0.9)		
HDL-C (mmol/l)	1.5 (0.4)	1.5 (0.4)	1.5 (0.4)	1.5 (0.4)		
Triglycerides (mmol/l)*	1.5 (1.0-2.1)	1.4 (1.0-2.0)	1.5 (1.1-2.1)	1.5 (1.1-2.0)		
Apolipoprotein-B (mmol/l)	1.0 (0.2)	1.0 (0.2)	1.0 (0.2)	1.0 (0.2)		
Other cardiometabolic markers						
Systolic blood pressure (mmHg)	137.7 (18.6)	136.0 (18.2)	138.9 (18.3)	138.5 (18.5)	139.6 (19.3)	138.0 (18.8)
Diastolic blood pressure (mmHg)	82.4 (10.2)	81.5 (9.9)	80.4 (9.7)	80.4 (9.8)	79.0 (10.3)	78.5 (10.3)
HbA1c (mmol/mol) †	35.8 (6.4)	35.3 (5.9)	36.3 (5.3)	35.7 (4.7)		
C-reactive protein (mg/l)*	1.3 (0.6-2.7)	1.1 (0.6-2.3)	1.2 (0.6-2.4)	1.1 (0.6-2.1)		
Gamma-GT (U/l)*	26.0 (18.4-40.3)	24.2 (17.4-36.6)	25.4 (18.4-38.3)	24.9 (18.0-35.8)		

*Median (interquartile range) are presented for right-skewed outcomes instead of mean (SD)
† For HbA1c N=346,606 at baseline and N=7,703 at 1st follow-up, and for the 24-hour dietary assessment subset n=24 933 at baseline and n=650 at 1st follow-up

Supplemental Table S3. Associations of BASELINE fruit consumption with cardiometabolic risk factors at BASELINE (2006-2010) in the subset with dietary data from the frequency questionnaire [a]

Outcome	Model	Mean (95% CI) for <1 serv/day	Mean (95% CI) for ≥3 serv/day	≥3 vs <1 servings/day Mean difference (95% CI)	P linear trend [b]
BMI (kg/m²)	**Age, sex**	27.6 (27.6, 27.7)	27.3 (27.3, 27.4)	-0.28 (-0.34, -0.22)	<0.001*
	+ sociodemographic and lifestyle factors [c]	27.4 (27.4, 27.5)	27.4 (27.4, 27.4)	-0.03 (-0.09, 0.02)	0.019
	**+ dietary factors** [d]	27.2 (27.2, 27.3)	27.5 (27.4, 27.5)	0.26 (0.20, 0.32)	0.057
WC (cm)	**Age, sex**	91.3 (91.2, 91.4)	89.6 (89.5, 89.7)	-1.72 (-1.86, -1.58)	<0.001*
	+ sociodemographic and lifestyle factors [c]	90.6 (90.5, 90.7)	89.8 (89.7, 89.9)	-0.83 (-0.97, -0.69)	0.032
	**+ dietary factors** [d]	90.0 (89.9, 90.2)	90.1 (90.0, 90.1)	0.05 (-0.10, 0.19)	0.274
	+ BMI	90.2 (90.2, 90.3)	89.7 (89.7, 89.8)	-0.52 (-0.59, -0.45)	0.432
Body fat (%)	**Age, sex**	32.0 (32.0, 32.1)	31.0 (31.0, 31.0)	-1.02 (-1.09, -0.94)	<0.001*
	+ sociodemographic and lifestyle factors [c]	31.7 (31.7, 31.8)	31.1 (31.1, 31.1)	-0.61 (-0.69, -0.54)	0.039
	**+ dietary factors** [d]	31.4 (31.3, 31.4)	31.3 (31.2, 31.3)	-0.10 (-0.18, -0.02)	0.446
	+ BMI	31.5 (31.4, 31.5)	31.1 (31.1, 31.1)	-0.39 (-0.44, -0.35)	0.323
LDL-C (mmol/l)	**Age, sex**	3.7 (3.7, 3.7)	3.6 (3.6, 3.6)	-0.11 (-0.12, -0.10)	<0.001*
	+ sociodemographic and lifestyle factors [c]	3.7 (3.7, 3.7)	3.6 (3.6, 3.6)	-0.10 (-0.11, -0.09)	0.925
	**+ dietary factors** [d]	3.7 (3.7, 3.7)	3.6 (3.6, 3.6)	-0.08 (-0.09, -0.07)	0.858
	+ BMI	3.7 (3.7, 3.7)	3.6 (3.6, 3.6)	-0.08 (-0.09, -0.07)	0.852
HDL-C (mmol/l)	**Age, sex**	1.5 (1.5, 1.5)	1.5 (1.4, 1.5)	-0.01 (-0.01, -0.00)	<0.001*
	+ sociodemographic and lifestyle factors [c]	1.5 (1.5, 1.5)	1.5 (1.5, 1.5)	-0.02 (-0.02, -0.02)	0.127
	**+ dietary factors** [d]	1.5 (1.5, 1.5)	1.4 (1.4, 1.4)	-0.04 (-0.04, -0.03)	0.870
	+ BMI	1.5 (1.5, 1.5)	1.4 (1.4, 1.5)	-0.03 (-0.04, -0.03)	0.419
Triglycerides (mmol/l) [e]	**Age, sex**	1.6 (1.6, 1.6)	1.5 (1.5, 1.5)	-7.4% (-8.0%, -6.8%)	<0.001*
	+ sociodemographic and lifestyle factors [c]	1.5 (1.5, 1.6)	1.5 (1.5, 1.5)	-3.9% (-4.5%, -3.4%)	0.848
	**+ dietary factors** [d]	1.5 (1.5, 1.5)	1.5 (1.5, 1.5)	-1.1% (-1.7%, -0.5%)	0.175
	+ BMI	1.5 (1.5, 1.5)	1.5 (1.5, 1.5)	-1.9% (-2.5%, -1.3%)	0.506
Apo-B (mmol/l)	**Age, sex**	1.1 (1.1, 1.1)	1.0 (1.0, 1.0)	-0.03 (-0.04, -0.03)	0.004*
	+ sociodemographic and lifestyle factors [c]	1.1 (1.1, 1.1)	1.0 (1.0, 1.0)	-0.03 (-0.03, -0.02)	0.913
	**+ dietary factors** [d]	1.1 (1.1, 1.1)	1.0 (1.0, 1.0)	-0.02 (-0.03, -0.02)	0.789
	+ BMI	1.1 (1.1, 1.1)	1.0 (1.0, 1.0)	-0.02 (-0.03, -0.02)	0.873
Systolic BP (mmHg)	**Age, sex**	138.4 (138.2, 138.6)	137.5 (137.4, 137.6)	-0.89 (-1.10, -0.69)	0.006*
	+ sociodemographic and lifestyle factors [c]	138.3 (138.1, 138.5)	137.6 (137.5, 137.7)	-0.65 (-0.86, -0.44)	0.002*
	**+ dietary factors** [d]	138.0 (137.8, 138.2)	137.7 (137.6, 137.8)	-0.28 (-0.50, -0.06)	0.016
	+ BMI	138.1 (137.9, 138.3)	137.6 (137.5, 137.7)	-0.45 (-0.67, -0.23)	0.062
Diastolic BP (mmHg)	**Age, sex**	83.1 (83.0, 83.2)	82.2 (82.2, 82.3)	-0.89 (-1.01, -0.77)	0.020*
	+ sociodemographic and lifestyle factors [c]	83.0 (82.9, 83.1)	82.3 (82.3, 82.4)	-0.70 (-0.83, -0.58)	0.001*
	**+ dietary factors** [d]	82.8 (82.7, 82.9)	82.4 (82.4, 82.5)	-0.35 (-0.48, -0.22)	0.124
	+ BMI	82.8 (82.7, 82.9)	82.3 (82.3, 82.4)	-0.50 (-0.63, -0.38)	0.376
HbA1c (mmol/mol)	**Age, sex**	36.1 (36.0, 36.1)	35.8 (35.8, 35.9)	-0.21 (-0.29, -0.13)	<0.001*

Supplemental Table S3. Associations of BASELINE fruit consumption with cardiometabolic risk factors at BASELINE (2006-2010) in the subset with dietary data from the frequency questionnaire [a]

Outcome	Model	Mean (95% CI) for <1 serv/day	Mean (95% CI) for ≥3 serv/day	≥3 vs <1 servings/day Mean difference (95% CI)	P linear trend [b]
	+ sociodemographic and lifestyle factors [c]	35.8 (35.7, 35.9)	35.9 (35.8, 35.9)	0.08 (0.00, 0.16)	0.011
	**+ dietary factors** [d]	35.6 (35.6, 35.7)	36.0 (35.9, 36.0)	0.34 (0.26, 0.43)	0.111
	+ BMI	35.7 (35.6, 35.7)	35.9 (35.9, 36.0)	0.27 (0.19, 0.35)	0.274
C-reactive protein (mg/l) [e]	**Age, sex**	1.7 (1.7, 1.7)	1.3 (1.3, 1.3)	-26.1% (-27.0%, -25.2%)	<0.001*
	+ sociodemographic and lifestyle factors [c]	1.6 (1.5, 1.6)	1.3 (1.3, 1.3)	-18.0% (-19.0%, -16.9%)	<0.001*
	**+ dietary factors** [d]	1.5 (1.5, 1.5)	1.3 (1.3, 1.3)	-10.0% (-11.2%, -8.8%)	0.004
	+ BMI	1.5 (1.5, 1.5)	1.3 (1.3, 1.3)	-12.2% (-13.3%, -11.2%)	0.029
Gamma-GT (U/l) [e]	**Age, sex**	32.4 (32.2, 32.6)	27.5 (27.4, 27.6)	-15.2% (-15.7%, -14.6%)	<0.001*
	+ sociodemographic and lifestyle factors [c]	31.2 (31.0, 31.4)	27.9 (27.8, 28.0)	-10.5% (-11.1%, -9.9%)	<0.001*
	**+ dietary factors** [d]	30.6 (30.4, 30.8)	28.2 (28.1, 28.3)	-7.8% (-8.4%, -7.1%)	<0.001*
	+ BMI	30.7 (30.5, 30.8)	28.0 (28.0, 28.1)	-8.5% (-9.2%, -7.9%)	<0.001*

Apo, apolipoprotein; BMI, body mass index; BP, blood pressure; CI, confidence interval; GT, glutamine transferase; HbA1c, haemoglobin A1c; HDL-C, high-density lipoprotein cholesterol; LDL-C, low-density lipoprotein cholesterol; WC, waist circumference
[a] n=365 534 for all the outcomes apart from HbA1c with n=346 606
[b] P-values that are still <0.05 after correction of false discovery rate based on the Benjamini-Hochberg method are denoted with an asterisk (*)
[c] Additionally adjusted for ethnicity, Townsend deprivation index, education, smoking, alcohol consumption, physical activity
[d] Additionally adjusted for dietary supplements and consumption of vegetables, spread type, non-oily fish, oily fish, red unprocessed meat, total processed meat, cheese, whole grains, refined grains, coffee, decafeinated coffee, and tea.
[e] Log-transformed variables are presented as %change rather than mean difference. Estimates were back-transformed with exponentiation.

Supplemental Table S4. Associations of BASELINE fruit consumption with cardiometabolic risk factors at FIRST FOLLOW-UP (2012-2013) in the subset with dietary data from the frequency questionnaire [a]

Outcome	Model	Mean (95% CI) for <1 serv/day	Mean (95% CI) for ≥3 serv/day	≥3 vs <1 servings/day Mean difference (95% CI)	P linear trend [b]
BMI (kg/m²)	**Age, sex**	27.6 (27.3, 27.9)	26.9 (26.8, 27.0)	-0.73 (-1.05, -0.41)	0.646
	+ sociodemographic and lifestyle factors [c]	27.4 (27.1, 27.7)	27.0 (26.8, 27.1)	-0.41 (-0.73, -0.09)	0.623
	**+ dietary factors** [d]	27.1 (26.8, 27.4)	27.0 (26.9, 27.1)	-0.09 (-0.42, 0.23)	0.522
WC (cm)	**Age, sex**	93.0 (92.3, 93.8)	90.2 (89.9, 90.6)	-2.77 (-3.60, -1.95)	0.491
	+ sociodemographic and lifestyle factors [c]	92.2 (91.5, 93.0)	90.4 (90.1, 90.8)	-1.78 (-2.61, -0.96)	0.916
	**+ dietary factors** [d]	91.6 (90.8, 92.3)	90.6 (90.3, 91.0)	-0.97 (-1.82, -0.13)	0.756
	+ BMI	91.1 (90.6, 91.6)	90.4 (90.2, 90.6)	-0.70 (-1.23, -0.17)	0.785
Body fat (%)	**Age, sex**	32.3 (31.9, 32.7)	30.7 (30.5, 30.9)	-1.58 (-2.02, -1.13)	0.301
	+ sociodemographic and lifestyle factors [c]	31.8 (31.4, 32.2)	30.8 (30.6, 31.0)	-1.03 (-1.48, -0.59)	0.842
	**+ dietary factors** [d]	31.4 (31.0, 31.8)	30.9 (30.7, 31.1)	-0.50 (-0.95, -0.05)	0.653
	+ BMI	31.2 (30.9, 31.4)	30.8 (30.7, 30.9)	-0.37 (-0.68, -0.06)	0.955
LDL-C (mmol/l)	**Age, sex**	3.6 (3.5, 3.7)	3.6 (3.5, 3.6)	-0.04 (-0.10, 0.03)	0.479
	+ sociodemographic and lifestyle factors [c]	3.6 (3.6, 3.7)	3.6 (3.5, 3.6)	-0.04 (-0.10, 0.02)	0.835
	**+ dietary factors** [d]	3.6 (3.5, 3.7)	3.6 (3.5, 3.6)	-0.03 (-0.10, 0.03)	0.942
	+ BMI	3.6 (3.5, 3.7)	3.6 (3.5, 3.6)	-0.03 (-0.10, 0.03)	0.943
HDL-C (mmol/l)	**Age, sex**	1.5 (1.5, 1.5)	1.5 (1.5, 1.5)	0.01 (-0.01, 0.04)	0.319
	+ sociodemographic and lifestyle factors [c]	1.5 (1.5, 1.5)	1.5 (1.5, 1.5)	0.00 (-0.02, 0.03)	0.947
	**+ dietary factors** [d]	1.5 (1.5, 1.6)	1.5 (1.5, 1.5)	-0.02 (-0.05, 0.01)	0.625
	+ BMI	1.5 (1.5, 1.6)	1.5 (1.5, 1.5)	-0.02 (-0.05, 0.00)	0.477
Triglycerides (mmol/l) [e]	**Age, sex**	1.6 (1.5, 1.6)	1.5 (1.5, 1.5)	-7.5% (-10.6%, -4.3%)	0.984
	+ sociodemographic and lifestyle factors [c]	1.6 (1.5, 1.6)	1.5 (1.5, 1.5)	-5.0% (-8.2%, -1.7%)	0.645
	**+ dietary factors** [d]	1.5 (1.5, 1.6)	1.5 (1.5, 1.5)	-2.7% (-6.1%, 0.9%)	0.756
	+ BMI	1.5 (1.5, 1.6)	1.5 (1.5, 1.5)	-2.3% (-5.6%, 1.1%)	0.871
Apo-B (mmol/l)	**Age, sex**	1.0 (1.0, 1.1)	1.0 (1.0, 1.0)	-0.02 (-0.03, 0.00)	0.469
	+ sociodemographic and lifestyle factors [c]	1.0 (1.0, 1.1)	1.0 (1.0, 1.0)	-0.01 (-0.03, 0.00)	0.690
	**+ dietary factors** [d]	1.0 (1.0, 1.1)	1.0 (1.0, 1.0)	-0.01 (-0.03, 0.01)	0.890
	+ BMI	1.0 (1.0, 1.1)	1.0 (1.0, 1.0)	-0.01 (-0.03, 0.01)	0.928
Systolic BP (mmHg)	**Age, sex**	140.4 (139.3, 141.6)	139.4 (138.8, 139.9)	-1.07 (-2.34, 0.19)	0.057
	+ sociodemographic and lifestyle factors [c]	140.4 (139.2, 141.5)	139.4 (138.9, 139.9)	-0.97 (-2.25, 0.31)	0.096
	**+ dietary factors** [d]	140.1 (138.9, 141.2)	139.4 (138.9, 140.0)	-0.64 (-1.98, 0.69)	0.318
	+ BMI	139.9 (138.8, 141.1)	139.4 (138.8, 139.9)	-0.57 (-1.89, 0.75)	0.265
Diastolic BP (mmHg)	**Age, sex**	81.8 (81.2, 82.5)	80.4 (80.1, 80.7)	-1.42 (-2.12, -0.73)	0.355
	+ sociodemographic and lifestyle factors [c]	81.8 (81.1, 82.4)	80.4 (80.1, 80.7)	-1.33 (-2.04, -0.63)	0.314
	**+ dietary factors** [d]	81.5 (80.9, 82.2)	80.5 (80.2, 80.8)	-1.05 (-1.78, -0.32)	0.605
	+ BMI	81.4 (80.8, 82.0)	80.4 (80.1, 80.7)	-0.98 (-1.69, -0.27)	0.464
HbA1c (mmol/mol)	**Age, sex**	36.3 (35.9, 36.8)	36.3 (36.1, 36.5)	-0.03 (-0.48, 0.42)	0.487

Supplemental Table S4. Associations of BASELINE fruit consumption with cardiometabolic risk factors at FIRST FOLLOW-UP (2012-2013) in the subset with dietary data from the frequency questionnaire [a]

Outcome	Model	Mean (95% CI) for <1 serv/day	Mean (95% CI) for ≥3 serv/day	≥3 vs <1 servings/day Mean difference (95% CI)	P linear trend [b]
	+ sociodemographic and lifestyle factors [c]	36.1 (35.7, 36.5)	36.3 (36.2, 36.5)	0.20 (-0.25, 0.66)	0.613
	**+ dietary factors** [d]	36.1 (35.6, 36.5)	36.4 (36.2, 36.6)	0.35 (-0.12, 0.81)	0.055
	+ BMI	36.0 (35.6, 36.4)	36.4 (36.2, 36.6)	0.39 (-0.07, 0.85)	0.060
C-reactive protein (mg/l) [e]	**Age, sex**	1.6 (1.5, 1.8)	1.2 (1.2, 1.2)	-27.9% (-32.9%, -22.5%)	0.232
	+ sociodemographic and lifestyle factors [c]	1.6 (1.5, 1.7)	1.2 (1.2, 1.2)	-22.6% (-28.0%, -16.8%)	0.532
	**+ dietary factors** [d]	1.5 (1.4, 1.6)	1.2 (1.2, 1.3)	-17.4% (-23.4%, -10.9%)	0.548
	+ BMI	1.5 (1.4, 1.6)	1.2 (1.2, 1.3)	-16.5% (-22.3%, -10.4%)	0.397
Gamma-GT (U/l) [e]	**Age, sex**	32.1 (30.9, 33.3)	26.9 (26.4, 27.3)	-16.3% (-19.7%, -12.9%)	0.717
	+ sociodemographic and lifestyle factors [c]	31.2 (30.1, 32.4)	27.1 (26.7, 27.6)	-13.1% (-16.6%, -9.5%)	0.791
	**+ dietary factors** [d]	30.8 (29.7, 32.0)	27.3 (26.8, 27.8)	-11.5% (-15.2%, -7.7%)	0.693
	+ BMI	30.6 (29.5, 31.8)	27.2 (26.8, 27.7)	-11.2% (-14.8%, -7.4%)	0.770

Apo, apolipoprotein; BMI, body mass index; BP, blood pressure; CI, confidence interval; GT, glutamine transferase; HbA1c, haemoglobin A1c; HDL-C, high-density lipoprotein cholesterol; LDL-C, low-density lipoprotein cholesterol; WC, waist circumference
[a]n=11 510 for all the outcomes apart from HbA1c with n=7703
[b]P-values that are still <0.05 after correction of false discovery rate based on the Benjamini-Hochberg method are denoted with an asterisk (*)
[c]Additionally adjusted for ethnicity, Townsend deprivation index, education, smoking, alcohol consumption, physical activity
[d]Additionally adjusted for dietary supplements and consumption of vegetables, spread type, non-oily fish, oily fish, red unprocessed meat, total processed meat, cheese, whole grains, refined grains, coffee, decafeinated coffee, and tea.
[e]Log-transformed variables are presented as %change rather than mean difference. Estimates were back-transformed with exponentiation.

Supplemental Table S5. Associations of BASELINE fruit consumption with cardiometabolic risk factors at SECOND FOLLOW-UP (2014-2023) in the subset with dietary data from the frequency questionnaire [a]

Outcome	Model	Mean (95% CI) for <1 serv/day	Mean (95% CI) for ≥3 serv/day	≥3 vs <1 servings/day Mean difference (95% CI)	P linear trend [b]
BMI (kg/m²)	**Age, sex**	27.3 (27.2, 27.5)	26.5 (26.4, 26.6)	-0.84 (-1.01, -0.66)	0.020*
	+ sociodemographic and lifestyle factors [c]	27.1 (27.0, 27.3)	26.6 (26.5, 26.7)	-0.54 (-0.71, -0.37)	0.213
	**+ dietary factors** [d]	26.9 (26.8, 27.1)	26.6 (26.6, 26.7)	-0.27 (-0.45, -0.10)	0.807
WC (cm)	**Age, sex**	91.0 (90.6, 91.4)	88.4 (88.2, 88.6)	-2.57 (-3.02, -2.13)	0.003*
	+ sociodemographic and lifestyle factors [c]	90.3 (89.9, 90.7)	88.6 (88.4, 88.8)	-1.68 (-2.12, -1.24)	0.044
	**+ dietary factors** [d]	89.7 (89.3, 90.1)	88.8 (88.6, 89.0)	-0.90 (-1.35, -0.45)	0.401
	+ BMI	89.4 (89.1, 89.6)	88.7 (88.5, 88.8)	-0.71 (-1.01, -0.40)	0.625
Body fat (%)	**Age, sex**	32.4 (32.2, 32.6)	30.7 (30.6, 30.8)	-1.68 (-1.93, -1.44)	0.026
	+ sociodemographic and lifestyle factors [c]	32.1 (31.9, 32.3)	30.9 (30.8, 31.0)	-1.21 (-1.45, -0.97)	0.240
	**+ dietary factors** [d]	31.7 (31.5, 31.9)	31.0 (30.9, 31.1)	-0.70 (-0.94, -0.45)	0.541
	+ BMI	31.5 (31.4, 31.7)	30.9 (30.8, 31.0)	-0.60 (-0.78, -0.42)	0.869
Systolic BP (mmHg)	**Age, sex**	140.1 (139.4, 140.7)	139.8 (139.5, 140.1)	-0.28 (-1.00, 0.43)	0.030
	+ sociodemographic and lifestyle factors [c]	140.0 (139.3, 140.6)	139.9 (139.5, 140.2)	-0.10 (-0.83, 0.62)	0.009
	**+ dietary factors** [d]	139.8 (139.1, 140.5)	139.9 (139.6, 140.2)	0.09 (-0.66, 0.85)	0.029
	+ BMI	139.7 (139.0, 140.4)	139.8 (139.5, 140.2)	0.15 (-0.60, 0.90)	0.035
Diastolic BP (mmHg)	**Age, sex**	79.4 (79.0, 79.7)	79.0 (78.8, 79.2)	-0.39 (-0.79, 0.01)	0.032
	+ sociodemographic and lifestyle factors [c]	79.3 (79.0, 79.7)	79.0 (78.8, 79.2)	-0.34 (-0.74, 0.07)	0.030
	**+ dietary factors** [d]	79.2 (78.8, 79.5)	79.1 (78.9, 79.2)	-0.11 (-0.52, 0.31)	0.122
	+ BMI	79.1 (78.7, 79.5)	79.0 (78.9, 79.2)	-0.06 (-0.47, 0.34)	0.148

BMI, body mass index; BP, blood pressure; CI, confidence interval; WC, waist circumference
[a]n=38 988
[b]P-values that are still <0.05 after correction of false discovery rate based on the Benjamini-Hochberg method are denoted with an asterisk (*)
[c]Additionally adjusted for ethnicity, Townsend deprivation index, education, smoking, alcohol consumption, physical activity
[d]Additionally adjusted for dietary supplements and consumption of vegetables, spread type, non-oily fish, oily fish, red unprocessed meat, total processed meat, cheese, whole grains, refined grains, coffee, decafeinated coffee, and tea.

Supplemental Table S6. Fully adjusted[a] mean differences in cardiometabolic risk factors at different time points in the subsets with dietary data from the frequency questionnaire, presented as changes in SD.[b]

	Baseline (2006-2010)					First FUP (2012-2013)					Second FUP (2014-2023)				
**Outcome**	**Low fruit**	**High fruit**	**Mean Difference (95% CI)**	**SD[d]**	**Diff/ SD[e]**	**Low fruit**	**High fruit**	**Mean Difference (95% CI)**	**SD**	**Diff/ SD**	**Low fruit**	**High fruit**	**Mean Difference (95% CI)**	**SD**	**Diff/ SD**
**BMI**	27.21	27.47	0.26 (0.20, 0.32)	4.7	0.06	27.10	27.01	-0.1 (-0.42, 0.23)	4.4	0.02	26.91	26.64	-0.27 (-0.45, -0.10)	4.4	0.06
**Waist**	90.03	90.07	0.05 (-0.10,0.19)	13.3	0.00	91.59	90.62	-0.97 (-1.82, -0.13)	13	0.07	89.70	88.80	-0.9 (-1.35, -0.45)	12.7	0.07
**Body fat %**	31.36	31.26	-0.1 (-0.18,-0.02)	8.5	0.01	31.43	30.92	-0.5 (-0.95, -0.05)	8.3	0.06	31.68	30.99	-0.7 (-0.94, -0.45)	8.2	0.08
**HDL-C**	1.48	1.45	-0.04 (-0.04,-0.03)	0.4	**0.10**	1.54	1.52	-0.02 (-0.05, 0.01)	0.4	0.05					
**LDL-C**	3.66	3.58	-0.08 (-0.09,-0.07)	0.8	**0.10**	3.60	3.57	-0.03 (-0.10, 0.03)	0.9	0.03					
**log-TGI[c]**	0.42	0.41	-0.01 (-0.02,-0.01)	0.5	0.02	0.43	0.40	-0.03 (-0.06, -0.01)	0.5	0.05					
**ApoB**	1.06	1.04	-0.02 (-0.03,-0.02)	0.2	**0.11**	1.04	1.03	-0.01 (-0.03, 0.01)	0.2	0.06					
**SBP**	138.03	137.74	-0.28 (-0.50, -0.06)	18.6	0.02	140.06	139.42	-0.64 (-1.98, 0.69)	18.3	0.04	139.80	139.89	0.09 (-0.66, 0.85)	19.3	0.00
**DBP**	82.77	82.43	-0.35 (-0.48,-0.22)	10.2	0.03	81.53	80.48	-1.05 (-1.78, -0.32)	9.7	**0.11**	79.16	79.06	-0.11 (-0.52, 0.31)	10.3	0.01
**HbA1c**	35.62	35.97	0.34 (0.26, 0.43)	6.4	0.05	36.17	36.42	0.25 (-0.12, 0.81)	5.4	0.05					
**log-GGT**	3.42	3.34	-0.08 (-0.09,-0.07)	0.6	**0.13**	3.43	3.31	-0.12 (-0.16, -0.08)	0.6	**0.20**					
**log-CRP**	0.39	0.28	-0.11 (-0.12,-0.09)	1.0	**0.11**	0.39	0.20	-0.19 (-0.27, -0.12)	1.0	**0.19**					

Apo, apolipoprotein; BMI, body mass index; CI, confidence interval; GT, glutamine transferase; HbA1c, haemoglobin A1c; HDL-C, high-density lipoprotein cholesterol; LDL-C, low-density lipoprotein cholesterol; TGI, triglycerides; WC, waist circumference.

[a] Adjusted for age, sex, ethnicity, Townsend deprivation index, education, smoking, alcohol consumption, physical activity, dietary supplements, and consumption of vegetables, spread type, non-oily fish, oily fish, red unprocessed meat, total processed meat, cheese, whole grains and refined grains.

[b] n=365 534 for all the outcomes apart from HbA1c with n=346 606.

[c] Estimates of log-transformed variables were back-transformed with exponentiation.

[d] SD is the standard deviation of the outcome variable.

[e] Diff/SD is the mean difference divided by the SD of the outcome variable; estimates greater than 0.1 SD are in bold.

Supplemental Table S7. Sensitivity analyses for associations of BASELINE fruit consumption with cardiometabolic risk factors at baseline (2006-2010) in the subset with dietary data from the frequency questionnaire

Outcome	Model	Mean (95% CI) for <1 serv/day	Mean (95% CI) for ≥3 serv/day	≥3 vs <1 servings/day Mean difference (95% CI)
**Subset of second follow-up[b]**				
BMI (kg/m^2)	Age, sex	27.2 (27.0, 27.3)	26.6 (26.5, 26.7)	-0.61 (-0.77, -0.45)
	+ sociodemographic and lifestyle factors[c]	27.0 (26.9, 27.2)	26.6 (26.6, 26.7)	-0.36 (-0.53, -0.20)
	+ dietary factors[d]	26.8 (26.7, 27.0)	26.7 (26.6, 26.8)	-0.10 (-0.26, 0.07)
Waist circumference (cm)	Age, sex	90.0 (89.6, 90.3)	87.8 (87.6, 87.9)	-2.22 (-2.64, -1.80)
	+ sociodemographic and lifestyle factors[c]	89.4 (89.1, 89.8)	88.0 (87.8, 88.1)	-1.45 (-1.87, -1.04)
	+ dietary factors[d]	88.9 (88.5, 89.3)	88.2 (88.0, 88.4)	-0.67 (-1.10, -0.25)
	+ BMI	88.5 (88.3, 88.7)	88.0 (88.0, 88.1)	-0.46 (-0.69, -0.24)
Body fat (%)	Age, sex	31.0 (30.8, 31.2)	29.6 (29.5, 29.7)	-1.42 (-1.66, -1.19)
	+ sociodemographic and lifestyle factors[c]	30.7 (30.5, 30.9)	29.7 (29.6, 29.8)	-1.00 (-1.23, -0.77)
	+ dietary factors[d]	30.3 (30.1, 30.6)	29.9 (29.8, 29.9)	-0.49 (-0.73, -0.26)
	+ BMI	30.1 (30.0, 30.3)	29.8 (29.7, 29.8)	-0.38 (-0.52, -0.25)
Systolic blood pressure (mmHg)	Age, sex	135.8 (135.3, 136.4)	135.1 (134.8, 135.4)	-0.73 (-1.36, -0.09)
	+ sociodemographic and lifestyle factors[c]	135.7 (135.1, 136.2)	135.2 (134.9, 135.5)	-0.47 (-1.11, 0.18)
	+ dietary factors[d]	135.6 (135.0, 136.2)	135.2 (135.0, 135.5)	-0.38 (-1.05, 0.29)
	+ BMI	135.5 (134.9, 136.1)	135.2 (134.9, 135.4)	-0.30 (-0.96, 0.35)
Diastolic blood pressure (mmHg)	Age, sex	82.5 (82.1, 82.8)	81.5 (81.4, 81.7)	-0.92 (-1.30, -0.55)
	+ sociodemographic and lifestyle factors[c]	82.3 (82.0, 82.7)	81.6 (81.5, 81.8)	-0.67 (-1.05, -0.30)
	+ dietary factors[d]	82.2 (81.8, 82.5)	81.7 (81.5, 81.9)	-0.47 (-0.86, -0.08)
	+ BMI	82.0 (81.7, 82.4)	81.6 (81.5, 81.8)	-0.40 (-0.78, -0.03)
**Subset without diabetes at baseline[b]**				
BMI (kg/m^2)	Age, sex	27.5 (27.4, 27.5)	27.1 (27.1, 27.2)	-0.34 (-0.40, -0.29)
	+ sociodemographic and lifestyle factors[c]	27.3 (27.2, 27.3)	27.2 (27.2, 27.2)	-0.10 (-0.16, -0.05)
	+ dietary factors[d]	27.1 (27.0, 27.1)	27.3 (27.2, 27.3)	0.19 (0.13, 0.25)
Waist circumference (cm)	Age, sex	90.8 (90.7, 91.0)	88.9 (88.9, 89.0)	-1.90 (-2.04, -1.76)
	+ sociodemographic and lifestyle factors[c]	90.2 (90.1, 90.3)	89.1 (89.1, 89.2)	-1.04 (-1.18, -0.90)
	+ dietary factors[d]	89.6 (89.5, 89.7)	89.4 (89.4, 89.5)	-0.16 (-0.31, -0.01)
	+ BMI	89.7 (89.7, 89.8)	89.2 (89.1, 89.2)	-0.58 (-0.65, -0.50)
Body fat (%)	Age, sex	32.0 (31.9, 32.0)	30.9 (30.8, 30.9)	-1.09 (-1.17, -1.02)
	+ sociodemographic and lifestyle factors[c]	31.7 (31.6, 31.7)	31.0 (30.9, 31.0)	-0.69 (-0.77, -0.61)
	+ dietary factors[d]	31.3 (31.2, 31.4)	31.1 (31.1, 31.2)	-0.18 (-0.26, -0.10)
	+ BMI	31.4 (31.3, 31.4)	31.0 (31.0, 31.0)	-0.39 (-0.44, -0.35)
LDL-C (mmol/l)	Age, sex	3.7 (3.7, 3.7)	3.6 (3.6, 3.6)	-0.10 (-0.11, -0.09)
	+ sociodemographic and lifestyle factors[c]	3.7 (3.7, 3.7)	3.6 (3.6, 3.6)	-0.08 (-0.09, -0.07)
	+ dietary factors[d]	3.7 (3.7, 3.7)	3.6 (3.6, 3.6)	-0.06 (-0.07, -0.05)
	+ BMI	3.7 (3.7, 3.7)	3.6 (3.6, 3.6)	-0.06 (-0.07, -0.05)
HDL-C (mmol/l)	Age, sex	1.5 (1.5, 1.5)	1.5 (1.5, 1.5)	-0.00 (-0.01, -0.00)

Supplemental Table S7. Sensitivity analyses for associations of BASELINE fruit consumption with cardiometabolic risk factors at baseline (2006-2010) in the subset with dietary data from the frequency questionnaire

Outcome	Model	Mean (95% CI) for <1 serv/day	Mean (95% CI) for ≥3 serv/day	≥3 vs <1 servings/day Mean difference (95% CI)
	+ sociodemographic and lifestyle factors[c]	1.5 (1.5, 1.5)	1.5 (1.5, 1.5)	-0.02 (-0.02, -0.01)
	+ dietary factors[d]	1.5 (1.5, 1.5)	1.5 (1.5, 1.5)	-0.04 (-0.04, -0.03)
	+ BMI	1.5 (1.5, 1.5)	1.5 (1.5, 1.5)	-0.03 (-0.03, -0.03)
Triglycerides (mmol/l)[e]	Age, sex	1.6 (1.6, 1.6)	1.5 (1.5, 1.5)	-7.8% (-8.4%, -7.2%)
	+ sociodemographic and lifestyle factors[c]	1.5 (1.5, 1.5)	1.5 (1.5, 1.5)	-4.3% (-4.9%, -3.8%)
	+ dietary factors[d]	1.5 (1.5, 1.5)	1.5 (1.5, 1.5)	-1.5% (-2.1%, -0.8%)
	+ BMI	1.5 (1.5, 1.5)	1.5 (1.5, 1.5)	-2.1% (-2.7%, -1.5%)
Apolipoprotein-B (mmol/l)	Age, sex	1.1 (1.1, 1.1)	1.0 (1.0, 1.0)	-0.03 (-0.03, -0.03)
	+ sociodemographic and lifestyle factors[c]	1.1 (1.1, 1.1)	1.0 (1.0, 1.0)	-0.02 (-0.03, -0.02)
	+ dietary factors[d]	1.1 (1.1, 1.1)	1.0 (1.0, 1.0)	-0.02 (-0.02, -0.01)
	+ BMI	1.1 (1.1, 1.1)	1.0 (1.0, 1.0)	-0.02 (-0.02, -0.02)
Systolic blood pressure (mmHg)	Age, sex	138.3 (138.1, 138.5)	137.4 (137.3, 137.5)	-0.92 (-1.13, -0.71)
	+ sociodemographic and lifestyle factors[c]	138.1 (137.9, 138.3)	137.5 (137.4, 137.6)	-0.66 (-0.88, -0.44)
	+ dietary factors[d]	137.9 (137.7, 138.1)	137.6 (137.5, 137.7)	-0.26 (-0.49, -0.03)
	+ BMI	137.9 (137.7, 138.1)	137.5 (137.4, 137.6)	-0.39 (-0.62, -0.17)
Diastolic blood pressure (mmHg)	Age, sex	83.2 (83.1, 83.3)	82.3 (82.2, 82.3)	-0.89 (-1.01, -0.77)
	+ sociodemographic and lifestyle factors[c]	83.0 (82.9, 83.2)	82.4 (82.3, 82.4)	-0.68 (-0.81, -0.56)
	+ dietary factors[d]	82.8 (82.7, 82.9)	82.5 (82.4, 82.5)	-0.31 (-0.44, -0.18)
	+ BMI	82.8 (82.7, 82.9)	82.4 (82.3, 82.4)	-0.43 (-0.56, -0.31)
HbA1c (mmol/mol)	Age, sex	35.5 (35.4, 35.5)	34.9 (34.9, 35.0)	-0.55 (-0.61, -0.49)
	+ sociodemographic and lifestyle factors[c]	35.3 (35.2, 35.3)	35.0 (34.9, 35.0)	-0.32 (-0.37, -0.26)
	+ dietary factors[d]	35.1 (35.1, 35.2)	35.1 (35.0, 35.1)	-0.09 (-0.15, -0.03)
	+ BMI	35.2 (35.1, 35.2)	35.0 (35.0, 35.1)	-0.12 (-0.18, -0.06)
C-reactive protein (mg/l)[e]	Age, sex	1.7 (1.7, 1.7)	1.2 (1.2, 1.2)	-26.6% (-27.6%, -25.7%)
	+ sociodemographic and lifestyle factors[c]	1.5 (1.5, 1.6)	1.3 (1.3, 1.3)	-18.4% (-19.5%, -17.4%)
	+ dietary factors[d]	1.5 (1.4, 1.5)	1.3 (1.3, 1.3)	-10.4% (-11.6%, -9.2%)
	+ BMI	1.5 (1.4, 1.5)	1.3 (1.3, 1.3)	-12.0% (-13.1%, -10.9%)
Gamma-GT (U/l)[e]	Age, sex	32.1 (31.9, 32.3)	27.1 (27.0, 27.2)	-15.6% (-16.2%, -15.0%)
	+ sociodemographic and lifestyle factors[c]	30.9 (30.7, 31.1)	27.5 (27.4, 27.6)	-11.1% (-11.7%, -10.4%)
	+ dietary factors[d]	30.3 (30.1, 30.5)	27.8 (27.7, 27.9)	-8.4% (-9.1%, -7.7%)
	+ BMI	30.4 (30.2, 30.6)	27.7 (27.6, 27.8)	-8.9% (-9.6%, -8.3%)
**Subset not taking lipid-lowering medication at baseline[b]**				
LDL-C (mmol/l)	Age, sex	3.8 (3.8, 3.8)	3.7 (3.7, 3.7)	-0.10 (-0.11, -0.09)
	+ sociodemographic and lifestyle factors[c]	3.8 (3.8, 3.8)	3.7 (3.7, 3.7)	-0.08 (-0.09, -0.07)
	+ dietary factors[d]	3.8 (3.7, 3.8)	3.7 (3.7, 3.7)	-0.06 (-0.07, -0.05)
	+ BMI	3.8 (3.8, 3.8)	3.7 (3.7, 3.7)	-0.07 (-0.08, -0.06)
HDL-C (mmol/l)	Age, sex	1.5 (1.5, 1.5)	1.5 (1.5, 1.5)	-0.00 (-0.01, -0.00)

Supplemental Table S7. Sensitivity analyses for associations of BASELINE fruit consumption with cardiometabolic risk factors at baseline (2006-2010) in the subset with dietary data from the frequency questionnaire

Outcome	Model	Mean (95% CI) for <1 serv/day	Mean (95% CI) for ≥3 serv/day	≥3 vs <1 servings/day Mean difference (95% CI)
	+ sociodemographic and lifestyle factors[c]	1.5 (1.5, 1.5)	1.5 (1.5, 1.5)	-0.02 (-0.02, -0.01)
	+ dietary factors[d]	1.5 (1.5, 1.5)	1.5 (1.5, 1.5)	-0.03 (-0.04, -0.03)
	+ BMI	1.5 (1.5, 1.5)	1.5 (1.5, 1.5)	-0.03 (-0.03, -0.03)
Triglycerides (mmol/l)[e]	Age, sex	1.6 (1.5, 1.6)	1.4 (1.4, 1.4)	-7.6% (-8.2%, -7.0%)
	+ sociodemographic and lifestyle factors[c]	1.5 (1.5, 1.5)	1.4 (1.4, 1.5)	-4.2% (-4.8%, -3.6%)
	+ dietary factors[d]	1.5 (1.5, 1.5)	1.5 (1.5, 1.5)	-1.3% (-2.0%, -0.7%)
	+ BMI	1.5 (1.5, 1.5)	1.5 (1.5, 1.5)	-2.0% (-2.7%, -1.4%)
Apolipoprotein-B (mmol/l)	Age, sex	1.1 (1.1, 1.1)	1.1 (1.1, 1.1)	-0.03 (-0.04, -0.03)
	+ sociodemographic and lifestyle factors[c]	1.1 (1.1, 1.1)	1.1 (1.1, 1.1)	-0.02 (-0.03, -0.02)
	+ dietary factors[d]	1.1 (1.1, 1.1)	1.1 (1.1, 1.1)	-0.02 (-0.02, -0.01)
	+ BMI	1.1 (1.1, 1.1)	1.1 (1.1, 1.1)	-0.02 (-0.02, -0.02)
**Subset not taking antihypertensive medication at baseline[b]**				
Systolic blood pressure (mmHg)	Age, sex	136.7 (136.5, 136.9)	135.7 (135.6, 135.9)	-0.91 (-1.14, -0.68)
	+ sociodemographic and lifestyle factors[c]	136.5 (136.3, 136.7)	135.8 (135.7, 135.9)	-0.65 (-0.88, -0.42)
	+ dietary factors[d]	136.2 (136.0, 136.4)	136.0 (135.9, 136.1)	-0.18 (-0.42, 0.06)
	+ BMI	136.2 (136.0, 136.4)	135.9 (135.8, 136.0)	-0.33 (-0.57, -0.09)
Diastolic blood pressure (mmHg)	Age, sex	82.6 (82.4, 82.7)	81.6 (81.5, 81.7)	-0.96 (-1.09, -0.83)
	+ sociodemographic and lifestyle factors[c]	82.4 (82.3, 82.5)	81.7 (81.6, 81.8)	-0.73 (-0.86, -0.60)
	+ dietary factors[d]	82.1 (82.0, 82.2)	81.8 (81.8, 81.9)	-0.31 (-0.45, -0.17)
	+ BMI	82.2 (82.1, 82.3)	81.7 (81.7, 81.8)	-0.45 (-0.58, -0.32)
**Subset with CRP<10 mg/l[b]**				
C-reactive protein (mg/l)[e]	Age, sex	1.5 (1.5, 1.5)	1.1 (1.1, 1.2)	-23.5% (-24.4%, -22.6%)
	+ sociodemographic and lifestyle factors[c]	1.4 (1.4, 1.4)	1.2 (1.2, 1.2)	-16.4% (-17.4%, -15.4%)
	+ dietary factors[d]	1.3 (1.3, 1.3)	1.2 (1.2, 1.2)	-9.1% (-10.2%, -8.0%)
	+ BMI	1.3 (1.3, 1.3)	1.2 (1.2, 1.2)	-11.2% (-12.1%, -10.2%)

[a]P-values that are still <0.05 after correction of false discovery rate based on the Benjamini-Hochberg method are denoted with an asterisk (*)
[b]Subset of second follow-up: n=38 988; Subset without diabetes at baseline: n=350 118; Subset not taking lipid-lowering medication at baseline: n=317 149; Subset not taking antihypertensive medication at baseline: n=301 222; Subset with CRP<10 mg/l: n=351 371
[c]Additionally adjusted for ethnicity, Townsend deprivation index, education, smoking, alcohol consumption, physical activity
[d]Additionally adjusted for dietary supplements and consumption of vegetables, spread type, non-oily fish, oily fish, red unprocessed meat, total processed meat, cheese, whole grains, refined grains, coffee, decafeinated coffee, and tea.
[e]Log-transformed variables are presented as %change rather than mean difference. Estimates were back-transformed with exponentiation

Supplemental Table S8. Cross-sectional associations of BASELINE fruit consumption with cardiometabolic risk factors at BASELINE (2009-2010) in the subset with dietary data from 2 to 5 WebQ 24-hour dietary assessments (2009-2012) [a]

		Mean (95% CI) for lowest and highest quintile of fruit intake		Q5 vs Q1 of fruit intake	
**Outcome**	**Model**	**Q1 0.4 (0-0.8) servings / day**	**Q5 4.4 (3.5-19.6) servings / day**	**Mean difference (95% CI)**	**P linear trend [b]**
BMI (kg/m^2)	**Age, sex**	**27.2 (27.1, 27.4)**	**26.7 (26.5, 26.8)**	**-0.6 (-0.8, -0.4)**	**<0.001***
	+ sociodemographic and lifestyle factors [c]	27.1 (27.0, 27.2)	26.8 (26.7, 26.9)	-0.3 (-0.5, -0.2)	<0.001*
	**+ dietary factors [d]**	**26.9 (26.8, 27.0)**	**26.9 (26.8, 27.1)**	**0.1 (-0.1, 0.3)**	**0.194**
Waist circumference (cm)	**Age, sex**	**90.1 (89.8, 90.4)**	**87.8 (87.5, 88.1)**	**-2.3 (-2.8, -1.9)**	**<0.001***
	+ sociodemographic and lifestyle factors [c]	89.6 (89.3, 89.9)	88.1 (87.8, 88.5)	-1.5 (-1.9, -1.0)	<0.001*
	**+ dietary factors [d]**	**89.1 (88.8, 89.5)**	**88.5 (88.2, 88.8)**	**-0.6 (-1.1, -0.2)**	**0.047**
	+ BMI	88.7 (88.6, 88.9)	87.9 (87.8, 88.1)	-0.8 (-1.0, -0.6)	<0.001*
Body fat (%)	**Age, sex**	**31.8 (31.7, 32.0)**	**30.2 (30.1, 30.4)**	**-1.6 (-1.8, -1.4)**	**<0.001***
	+ sociodemographic and lifestyle factors [c]	31.6 (31.4, 31.8)	30.4 (30.3, 30.6)	-1.2 (-1.4, -0.9)	<0.001*
	**+ dietary factors [d]**	**31.3 (31.1, 31.4)**	**30.7 (30.5, 30.9)**	**-0.6 (-0.8, -0.3)**	**<0.001***
	+ BMI	31.0 (30.9, 31.1)	30.4 (30.3, 30.5)	-0.6 (-0.8, -0.5)	<0.001*
LDL-C (mmol/l)	**Age, sex**	**3.63 (3.60, 3.65)**	**3.55 (3.53, 3.57)**	**-0.07 (-0.11, -0.04)**	**<0.001***
	+ sociodemographic and lifestyle factors [c]	3.62 (3.60, 3.64)	3.56 (3.53, 3.58)	-0.06 (-0.10, -0.03)	<0.001*
	**+ dietary factors [d]**	**3.61 (3.59, 3.64)**	**3.56 (3.54, 3.58)**	**-0.05 (-0.09, -0.02)**	**0.002***
	+ BMI	3.61 (3.59, 3.63)	3.56 (3.53, 3.58)	-0.05 (-0.09, -0.02)	0.002*
HDL-C (mmol/l)	**Age, sex**	**1.50 (1.50, 1.51)**	**1.49 (1.49, 1.50)**	**-0.01 (-0.02, 0.00)**	**0.092**
	+ sociodemographic and lifestyle factors [c]	1.50 (1.49, 1.50)	1.50 (1.49, 1.51)	0.01 (-0.01, 0.02)	0.569
	**+ dietary factors [d]**	**1.50 (1.49, 1.51)**	**1.50 (1.49, 1.51)**	**-0.01 (-0.02, 0.01)**	**0.211**
	+ BMI	1.51 (1.50, 1.52)	1.50 (1.49, 1.51)	-0.00 (-0.02, 0.01)	0.392
Triglycerides (mmol/l)[e]	**Age, sex**	**1.51 (1.49, 1.53)**	**1.40 (1.38, 1.42)**	**-6.9% (-8.6%, -5.2%)**	**<0.001***
	+ sociodemographic and lifestyle factors [c]	1.50 (1.48, 1.52)	1.41 (1.40, 1.43)	-5.7% (-7.4%, -3.9%)	<0.001*
	**+ dietary factors [d]**	**1.49 (1.47, 1.51)**	**1.43 (1.41, 1.44)**	**-4.4% (-6.2%, -2.5%)**	**<0.001***
	+ BMI	1.50 (1.48, 1.52)	1.43 (1.41, 1.45)	-4.6% (-6.3%, -2.8%)	<0.001*
Apo-B (mmol/l)	**Age, sex**	**1.05 (1.04, 1.05)**	**1.03 (1.02, 1.03)**	**-0.02 (-0.03, -0.01)**	**<0.001***
	+ sociodemographic and lifestyle factors [c]	1.05 (1.04, 1.05)	1.03 (1.02, 1.03)	-0.02 (-0.03, -0.01)	<0.001*
	**+ dietary factors [d]**	**1.04 (1.04, 1.05)**	**1.03 (1.02, 1.04)**	**-0.01 (-0.02, -0.01)**	**0.003***
	+ BMI	1.04 (1.04, 1.05)	1.03 (1.02, 1.03)	-0.02 (-0.02, -0.01)	0.002*
Systolic blood pressure (mmHg)	**Age, sex**	**136.9 (136.4, 137.3)**	**136.0 (135.5, 136.4)**	**-0.9 (-1.6, -0.3)**	**0.009***
	+ sociodemographic and lifestyle factors [c]	136.6 (136.1, 137.0)	136.2 (135.8, 136.7)	-0.3 (-1.0, 0.3)	0.498
	**+ dietary factors [d]**	**136.3 (135.9, 136.8)**	**136.4 (135.9, 136.8)**	**0.1 (-0.6, 0.7)**	**0.573**
	+ BMI	136.2 (135.7, 136.6)	136.2 (135.7, 136.6)	0.0 (-0.7, 0.7)	0.759

Supplemental Table S8. Cross-sectional associations of BASELINE fruit consumption with cardiometabolic risk factors at BASELINE (2009-2010) in the subset with dietary data from 2 to 5 WebQ 24-hour dietary assessments (2009-2012) [a]

		Mean (95% CI) for lowest and highest quintile of fruit intake		Q5 vs Q1 of fruit intake	
**Outcome**	**Model**	**Q1 0.4 (0-0.8) servings / day**	**Q5 4.4 (3.5-19.6) servings / day**	**Mean difference (95% CI)**	**P linear trend [b]**
Diastolic blood pressure (mmHg)	**Age, sex**	**82.3 (82.0, 82.6)**	**81.2 (81.0, 81.5)**	**-1.1 (-1.5, -0.7)**	**<0.001***
	+ sociodemographic and lifestyle factors [c]	82.0 (81.8, 82.3)	81.4 (81.2, 81.7)	-0.6 (-1.0, -0.2)	0.002*
	**+ dietary factors [d]**	**81.8 (81.5, 82.1)**	**81.6 (81.3, 81.9)**	**-0.2 (-0.6, 0.2)**	**0.471**
	+ BMI	81.7 (81.4, 81.9)	81.4 (81.2, 81.7)	-0.3 (-0.6, 0.1)	0.245
HbA1c (mmol/mol)	**Age, sex**	**35.5 (35.3, 35.6)**	**35.4 (35.2, 35.6)**	**-0.1 (-0.3, 0.2)**	**0.991**
	+ sociodemographic and lifestyle factors [c]	35.4 (35.2, 35.6)	35.4 (35.3, 35.6)	0.0 (-0.2, 0.2)	0.508
	**+ dietary factors [d]**	**35.3 (35.1, 35.5)**	**35.5 (35.3, 35.7)**	**0.2 (-0.0, 0.5)**	**0.017***
	+ BMI	35.3 (35.1, 35.4)	35.4 (35.3, 35.6)	0.2 (-0.0, 0.4)	0.031*
C-reactive protein (mg/l)[e]	**Age, sex**	**1.41 (1.37, 1.45)**	**1.09 (1.06, 1.12)**	**-23.1% (-26.1%, -20.0%)**	**<0.001***
	+ sociodemographic and lifestyle factors [c]	1.38 (1.35, 1.42)	1.12 (1.09, 1.15)	-19.0% (-22.2%, -15.7%)	<0.001*
	**+ dietary factors [d]**	**1.33 (1.29, 1.37)**	**1.18 (1.14, 1.21)**	**-11.6% (-15.2%, -7.9%)**	**<0.001***
	+ BMI	1.48 (1.45, 1.52)	1.30 (1.27, 1.34)	-12.2% (-15.4%, -8.9%)	<0.001*
Gamma-GT (U/l)[e]	**Age, sex**	**29.9 (29.4, 30.3)**	**25.9 (25.5, 26.3)**	**-13.3% (-15.1%, -11.4%)**	**<0.001***
	+ sociodemographic and lifestyle factors [c]	29.1 (28.7, 29.5)	26.7 (26.3, 27.1)	-8.3% (-10.2%, -6.4%)	<0.001*
	**+ dietary factors [d]**	**28.7 (28.3, 29.1)**	**27.1 (26.6, 27.5)**	**-5.8% (-7.8%, -3.7%)**	**<0.001***
	+ BMI	28.8 (28.4, 29.2)	27.1 (26.7, 27.5)	-6.0% (-8.0%, -4.0%)	<0.001*

Apo, apolipoprotein; BMI, body mass index; CI, confidence interval; Gamma-GT, Gamma-glutamyl Transferase; HDL-C, high density lipoprotein cholesterol; LDL-C, low density lipoprotein cholesterol; Q, quintile

[a] n=26 596 for all outcomes apart from HbA1c with n=24 933

[b] P-values that are still <0.05 after correction of false discovery rate based on the Benjamini-Hochberg method are denoted with an asterisk (*)

[c] Additionally adjusted for ethnicity, Townsend deprivation index, education, smoking, alcohol consumption and physical activity

[d] Additionally adjusted for dietary supplements and consumption of vegetables, wholegrains, refined grains, red meat, processed meat, non-oily fish, oily fish, butter, cheese, coffee, decaffeinated coffee, tea and total energy intake

[e] Log transformed variables are presented as % change rather than mean difference; Estimates were back transformed with exponentiation.

Rows in bold highlight the models presented in the supplemental figures

Supplemental Table S9. Prospective associations of BASELINE fruit consumption with cardiometabolic risk factors at FIRST FOLLOW-UP (2012-2013) in the subset with dietary data from 2 to 5 WebQ 24-hour dietary assessments (2009-2012) [a]

Outcome	Model	Mean (95% CI) for lowest and highest quintile of fruit intake<br>Q1<br>0.4 (0 - 0.8) servings / day	Q5<br>4.4 (3.5 - 19.6) servings / day	Q5 vs Q1 of fruit intake<br>Mean difference (95% CI)	P linear trend [b]
BMI (kg/m^2)	**Age, sex**	26.7 (26.1, 27.3)	26.6 (26.1, 27.1)	-0.1 (-0.9, 0.7)	0.598
	+ sociodemographic and lifestyle factors [c]	26.5 (25.9, 27.1)	26.7 (26.1, 27.2)	0.1 (-0.7, 0.9)	0.410
	**+ dietary factors [d]**	26.3 (25.7, 26.9)	26.8 (26.3, 27.3)	0.5 (-0.3, 1.3)	0.049
Waist circumference (cm)	**Age, sex**	91.2 (89.7, 92.8)	89.3 (87.9, 90.7)	-1.9 (-4.0, 0.2)	0.535
	+ sociodemographic and lifestyle factors [c]	90.7 (89.1, 92.3)	89.6 (88.2, 91.0)	-1.1 (-3.2, 1.0)	0.993
	**+ dietary factors [d]**	90.1 (88.6, 91.7)	89.9 (88.5, 91.3)	-0.3 (-2.4, 1.9)	0.372
	+ baseline BMI	90.7 (89.7, 91.8)	89.0 (88.1, 89.9)	-1.8 (-3.2, -0.4)	0.182
Body fat (%)	**Age, sex**	31.2 (30.4, 32.1)	29.8 (29.0, 30.6)	-1.5 (-2.6, -0.3)	0.038*
	+ sociodemographic and lifestyle factors [c]	31.0 (30.1, 31.8)	30.0 (29.2, 30.7)	-1.0 (-2.2, 0.2)	0.131
	**+ dietary factors [d]**	30.6 (29.7, 31.5)	30.2 (29.4, 31.0)	-0.4 (-1.6, 0.8)	0.822
	+ baseline BMI	30.9 (30.3, 31.5)	29.7 (29.2, 30.3)	-1.2 (-2.0, -0.3)	0.009*
LDL-C (mmol/l)	**Age, sex**	3.62 (3.49, 3.74)	3.55 (3.43, 3.66)	-0.07 (-0.24, 0.10)	0.332
	+ sociodemographic and lifestyle factors [c]	3.62 (3.49, 3.75)	3.55 (3.43, 3.66)	-0.07 (-0.25, 0.10)	0.388
	**+ dietary factors [d]**	3.62 (3.49, 3.76)	3.55 (3.43, 3.67)	-0.08 (-0.26, 0.11)	0.481
	+ baseline BMI	3.62 (3.49, 3.76)	3.55 (3.43, 3.67)	-0.08 (-0.26, 0.11)	0.479
HDL-C (mmol/l)	**Age, sex**	1.53 (1.48, 1.58)	1.52 (1.48, 1.57)	-0.01 (-0.07, 0.06)	0.867
	+ sociodemographic and lifestyle factors [c]	1.53 (1.48, 1.58)	1.52 (1.48, 1.57)	-0.01 (-0.08, 0.06)	0.738
	**+ dietary factors [d]**	1.54 (1.49, 1.59)	1.52 (1.47, 1.56)	-0.02 (-0.09, 0.05)	0.552
	+ baseline BMI	1.53 (1.48, 1.58)	1.53 (1.49, 1.57)	-0.00 (-0.07, 0.07)	0.914
Triglycerides (mmol/l)[e]	**Age, sex**	1.63 (1.52, 1.74)	1.44 (1.36, 1.53)	-11.3% (-19.0%, -2.9%)	0.032*
	+ sociodemographic and lifestyle factors [c]	1.61 (1.50, 1.72)	1.46 (1.38, 1.55)	-9.2% (-17.2%, -0.5%)	0.098
	**+ dietary factors [d]**	1.61 (1.50, 1.72)	1.48 (1.38, 1.57)	-8.2% (-16.6%, 1.0%)	0.201
	+ baseline BMI	1.63 (1.52, 1.74)	1.47 (1.38, 1.55)	-10.2% (-18.2%, -1.5%)	0.055
Apo-B (mmol/l)	**Age, sex**	1.04 (1.01, 1.07)	1.01 (0.98, 1.04)	-0.03 (-0.07, 0.02)	0.272
	+ sociodemographic and lifestyle factors [c]	1.04 (1.00, 1.07)	1.02 (0.99, 1.05)	-0.02 (-0.07, 0.03)	0.390
	**+ dietary factors [d]**	1.04 (1.00, 1.08)	1.02 (0.98, 1.05)	-0.02 (-0.07, 0.02)	0.435
	+ baseline BMI	1.04 (1.00, 1.08)	1.01 (0.98, 1.05)	-0.03 (-0.08, 0.02)	0.380
Systolic blood pressure (mmHg)	**Age, sex**	137.1 (134.5, 139.6)	142.6 (140.4, 144.9)	5.6 (2.1, 9.0)	<0.001*
	+ sociodemographic and lifestyle factors [c]	136.8 (134.2, 139.4)	143.0 (140.7, 145.2)	6.2 (2.7, 9.7)	<0.001*
	**+ dietary factors [d]**	136.1 (133.5, 138.7)	143.7 (141.4, 146.0)	7.5 (4.0, 11.1)	<0.001*
	+ baseline BMI	136.3 (133.7, 138.9)	143.3 (141.0, 145.6)	7.0 (3.5, 10.5)	<0.001*

Supplemental Table S9. Prospective associations of BASELINE fruit consumption with cardiometabolic risk factors at FIRST FOLLOW-UP (2012-2013) in the subset with dietary data from 2 to 5 WebQ 24-hour dietary assessments (2009-2012) [a]

		Mean (95% CI) for lowest and highest quintile of fruit intake		Q5 vs Q1 of fruit intake	
**Outcome**	**Model**	**Q1 0.4 (0 - 0.8) servings / day**	**Q5 4.4 (3.5 - 19.6) servings / day**	**Mean difference (95% CI)**	**P linear trend [b]**
Diastolic blood pressure (mmHg)	**Age, sex**	80.3 (78.9, 81.8)	81.7 (80.4, 82.9)	1.3 (-0.6, 3.2)	0.034*
	+ sociodemographic and lifestyle factors [c]	80.2 (78.8, 81.6)	81.8 (80.5, 83.0)	1.6 (-0.4, 3.5)	0.022*
	**+ dietary factors [d]**	79.8 (78.4, 81.3)	82.0 (80.7, 83.3)	2.2 (0.2, 4.2)	0.003*
	+ baseline BMI	80.0 (78.5, 81.4)	81.8 (80.5, 83.1)	1.8 (-0.1, 3.8)	0.011*
HbA1c (mmol/mol)	**Age, sex**	35.7 (34.8, 36.6)	35.1 (34.3, 35.9)	-0.6 (-1.8, 0.6)	0.274
	+ sociodemographic and lifestyle factors [c]	35.7 (34.8, 36.6)	35.1 (34.3, 35.9)	-0.6 (-1.8, 0.7)	0.301
	**+ dietary factors [d]**	35.6 (34.6, 36.5)	35.2 (34.4, 36.0)	-0.4 (-1.7, 0.9)	0.572
	+ baseline BMI	35.7 (34.7, 36.6)	35.1 (34.3, 36.0)	-0.5 (-1.8, 0.8)	0.363
C-reactive protein (mg/l) [e]	**Age, sex**	1.24 (1.07, 1.42)	1.18 (1.03, 1.32)	-5.5% (-21.8%, 14.4%)	0.983
	+ sociodemographic and lifestyle factors [c]	1.22 (1.04, 1.40)	1.21 (1.05, 1.36)	-1.1% (-18.6%, 20.1%)	0.719
	**+ dietary factors [d]**	1.19 (1.02, 1.36)	1.27 (1.10, 1.43)	6.5% (-12.9%, 30.3%)	0.194
	+ baseline BMI	1.28 (1.10, 1.46)	1.29 (1.12, 1.45)	0.5% (-17.0%, 21.8%)	0.551
Gamma-GT (U/l) [e]	**Age, sex**	28.50 (26.34, 30.66)	26.45 (24.65, 28.24)	-7.2% (-16.2%, 2.7%)	0.157
	+ sociodemographic and lifestyle factors [c]	27.90 (25.81, 30.00)	27.04 (25.22, 28.85)	-3.1% (-12.5%, 7.3%)	0.455
	**+ dietary factors [d]**	27.31 (25.25, 29.38)	27.93 (26.01, 29.85)	2.3% (-7.9%, 13.6%)	0.596
	+ baseline BMI	27.61 (25.54, 29.67)	27.74 (25.87, 29.62)	0.5% (-9.4%, 11.5%)	0.908

Apo, apolipoprotein; BMI, body mass index; CI, confidence interval; Gamma-GT, Gamma-glutamyl Transferase; HDL-C, high density lipoprotein cholesterol; LDL-C, low density lipoprotein cholesterol; Q, quintile

[a] n=1092 for all outcomes apart from HbA1c with n=650

[b] P-values that are still <0.05 after correction of false discovery rate based on the Benjamini-Hochberg method are denoted with an asterisk (*)

[c] Additionally adjusted for ethnicity, Townsend deprivation index, education, smoking, alcohol consumption and physical activity

[d] Additionally adjusted for dietary supplements and consumption of vegetables, wholegrains, refined grains, red meat, processed meat, non-oily fish, oily fish, butter, cheese, coffee, decaffeinated coffee, tea and total energy intake

[e] Log transformed variables: Beta coefficients and confidence intervals are presented as % change rather than mean difference. Estimated means and confidence intervals were back-transformed with exponentiation.

Rows in bold highlight the models presented in the supplemental figures.

Supplemental Table S10. Prospective association of BASELINE fruit consumption with cardiometabolic risk factors at SECOND FOLLOW-UP (2014-2023) in the subset with dietary data from 2 to 5 WebQ 24-hour dietary assessments (2009-2012) [a]

Outcome	Model	Mean (95% CI) for lowest and highest quintile of fruit intake: Q1 0.4 (0-0.8) servings / day	Q5 4.4 (3.5-19.6) servings / day	Q5 vs Q1 of fruit intake: Mean difference (95% CI)	P linear trend [b]
BMI (kg/m^2)	**Age, sex**	26.9 (26.6, 27.2)	26.2 (25.9, 26.6)	-0.6 (-1.1, -0.2)	0.003*
	+ sociodemographic and lifestyle factors [c]	26.7 (26.4, 27.0)	26.4 (26.1, 26.7)	-0.3 (-0.8, 0.1)	0.151
	**+ dietary factors [d]**	26.5 (26.2, 26.8)	26.5 (26.2, 26.8)	-0.0 (-0.5, 0.5)	0.739
Waist circumference (cm)	**Age, sex**	89.1 (88.3, 89.9)	86.8 (86.0, 87.6)	-2.3 (-3.5, -1.1)	<0.001*
	+ sociodemographic and lifestyle factors [c]	88.6 (87.8, 89.4)	87.3 (86.5, 88.1)	-1.4 (-2.5, -0.2)	0.027*
	**+ dietary factors [d]**	88.2 (87.4, 89.1)	87.5 (86.7, 88.3)	-0.7 (-1.9, 0.5)	0.403
	+ BMI	88.1 (87.6, 88.7)	86.8 (86.3, 87.4)	-1.3 (-2.1, -0.5)	0.005*
Body fat (%)	**Age, sex**	32.0 (31.6, 32.5)	29.9 (29.4, 30.3)	-2.1 (-2.8, -1.5)	<0.001*
	+ sociodemographic and lifestyle factors [c]	31.7 (31.3, 32.2)	30.1 (29.7, 30.5)	-1.6 (-2.3, -1.0)	<0.001*
	**+ dietary factors [d]**	31.4 (30.9, 31.8)	30.4 (29.9, 30.8)	-1.0 (-1.6, -0.3)	0.014*
	+ BMI	31.3 (31.0, 31.6)	30.0 (29.7, 30.4)	-1.3 (-1.7, -0.8)	<0.001*
Systolic blood pressure (mmHg)	**Age, sex**	138.5 (137.2, 139.8)	138.2 (136.9, 139.5)	-0.3 (-2.1, 1.5)	0.813
	+ sociodemographic and lifestyle factors [c]	138.3 (137.0, 139.7)	138.4 (137.1, 139.6)	0.0 (-1.8, 1.9)	0.561
	**+ dietary factors [d]**	138.0 (136.7, 139.4)	138.6 (137.3, 140.0)	0.6 (-1.3, 2.6)	0.246
	+ BMI	138.0 (136.7, 139.3)	138.4 (137.1, 139.6)	0.4 (-1.5, 2.3)	0.363
Diastolic blood pressure (mmHg)	**Age, sex**	79.3 (78.6, 80.1)	78.1 (77.3, 78.8)	-1.3 (-2.3, -0.2)	0.043*
	+ sociodemographic and lifestyle factors [c]	79.2 (78.4, 79.9)	78.1 (77.4, 78.9)	-1.1 (-2.1, 0.0)	0.107
	**+ dietary factors [d]**	79.0 (78.3, 79.8)	78.3 (77.5, 79.0)	-0.8 (-1.9, 0.3)	0.285
	+ BMI	79.0 (78.3, 79.7)	78.1 (77.3, 78.8)	-0.9 (-2.0, 0.1)	0.150

BMI, body mass index; CI, confidence interval; Q, quintile

[a] n=3730

[b] P-values that are still <0.05 after correction of false discovery rate based on the Benjamini-Hochberg method are denoted with an asterisk (*)

[c] Additionally adjusted for ethnicity, Townsend deprivation index, education, smoking, alcohol consumption and physical activity

[d] Additionally adjusted for dietary supplements and consumption of vegetables, wholegrains, refined grains, red meat, processed meat, non-oily fish, oily fish, butter, cheese, coffee, decaffeinated coffee, tea and total energy intake

Rows in bold highlight the models presented in the supplemental figures.

Supplemental Table S11. Summary of associations between fruit intake at baseline and cardiometabolic risk factors at all time-points, by method of fruit intake measurement, comparing ~3 vs <1 servings per day

Outcome	Frequency questionnaire ≥3 vs <1 serving / day Mean difference (95% CI) Further adjusted model [b]	24-h dietary assessment Fourth vs first quintile [a] of fruit intake Mean difference (95% CI) Further adjusted model [b]	Further adjusted model plus total energy intake
**BASELINE**	**N= 365 534 (N_{HbA1c} = 346 606)**	**N = 26 596 (N_{HbA1c} = 24 933)**	
BMI (kg/m²)	0.26 (0.20, 0.32)	-0.26 (-0.43, -0.09)	-0.27 (-0.45, -0.10)
Waist circumference (cm)	0.05 (-0.10, 0.19)	-1.09 (-1.53, -0.65)	-1.18 (-1.62, -0.74)
Body fat (%)	-0.10 (-0.18,-0.02)	-0.62 (-0.86, -0.37)	-0.60 (-0.84, -0.36)
LDL-C (mmol/l)	-0.08 (-0.09, -0.07)	-0.04 (-0.07, -0.00)	-0.04 (-0.07, -0.01)
HDL-C (mmol/l)	-0.04 (-0.04, -0.03)	0.01 (-0.01, 0.02)	0.01 (-0.00, 0.02)
Triglycerides (mmol/l) [c]	-1.1% (-1.7%, -0.5%)	-2.6% (-4.4%, -0.8%)	-3.1% (-5.0%, -1.3%)
Apo-B (mmol/l)	-0.02 (-0.03, -0.02)	-0.01 (-0.02, -0.00)	-0.01 (-0.02, -0.00)
Systolic blood pressure (mmHg)	-0.28 (-0.50, -0.06)	-0.54 (-1.20, 0.12)	-0.50 (-1.16, 0.16)
Diastolic blood pressure (mmHg)	-0.35 (-0.48, -0.22)	-0.34 (-0.72, 0.04)	-0.33 (-0.71, 0.06)
HbA1c (mmol/mol)	0.34 (0.26, 0.43)	0.07 (-0.16, 0.30)	0.06 (-0.17, 0.30)
C-reactive protein (mg/l) [c]	-10.0% (-11.2%, -8.8%)	-10.8% (-14.3%, -7.1%)	-11.0% (-14.5%, -7.4%)
Gamma-GT (U/l) [c]	-7.8% (-8.4%, -7.1%)	-6.8% (-8.8%, -4.8%)	-6.7% (-8.6%, -4.7%)
**FIRST FOLLOW-UP**	**N = 11 510 (N_{HbA1c} = 7703)**	**N = 1092 (N_{HbA1c} = 650)**	
BMI (kg/m²)	-0.09 (-0.42, 0.23)	0.22 (-0.58, 1.02)	0.19 (-0.61, 0.99)
Waist circumference (cm)	-0.97 (-1.82, -0.13)	-0.19 (-2.27, 1.89)	-0.35 (-2.43, 1.73)
Body fat (%)	-0.50 (-0.95, -0.05)	-0.51 (-1.68, 0.65)	-0.54 (-1.71, 0.63)
LDL-C (mmol/l)	-0.03 (-0.10, 0.03)	0.02 (-0.16, 0.19)	0.01 (-0.16, 0.19)
HDL-C (mmol/l)	-0.02 (-0.05, 0.01)	0.01 (-0.06, 0.08)	0.02 (-0.05, 0.09)
Triglycerides (mmol/l)[d]	-2.7% (-6.1%, 0.9%)	-9.9% (-17.8%, -1.2%)	-10.3% (-18.3%, -1.6%)
Apo-B (mmol/l)	-0.01 (-0.03, 0.01)	0.00 (-0.04, 0.05)	0.00 (-0.04, 0.05)
Systolic blood pressure (mmHg)	-0.64, (-1.98, 0.69)	1.84 (-1.62, 5.30)	1.92 (-1.55, 5.39)
Diastolic blood pressure (mmHg)	-1.05 (-1.78, -0.32)	0.95 (-0.98, 2.88)	0.96 (-0.98, 2.89)
HbA1c (mmol/mol)	0.35 (-0.12, 0.81)	0.12 (-1.13, 1.36)	0.12 (-1.12, 1.37)
C-reactive protein (mg/l) [d]	-17.4% (-23.4%, -10.9%)	1.6% (-16.3%, 23.4%)	1.2% (-16.7%, 23.0%)
Gamma-GT (U/l) [d]	-11.5% (-15.2%, -7.7%)	-1.1% (-10.6%, 9.5%)	-0.4% (-10.1%, 10.2%)
**SECOND FOLLOW-UP**	**N = 38 988**	**N = 3730**	
BMI (kg/m²)	-0.27 (-0.45, -0.10)	-0.18 (-0.62, 0.26)	-0.20 (-0.64, 0.24)
Waist circumference (cm)	-0.90 (-1.35, -0.45)	-0.74 (-1.89, 0.41)	-0.83 (-1.98, 0.33)
Body fat (%)	-0.70 (-0.94, -0.45)	-0.82 (-1.45, -0.18)	-0.82 (-1.45, -0.18)
Systolic blood pressure (mmHg)	0.09 (-0.66, 0.85)	0.17 (-1.70, 2.04)	0.22 (-1.65, 2.09)
Diastolic blood pressure (mmHg)	-0.11 (-0.52, 0.31)	-0.81 (-1.87, 0.25)	-0.82 (-1.89, 0.24)

Apo, apolipoprotein; BMI, body mass index; CI, confidence interval; Gamma-GT, Gamma-glutamyl Transferase; HDL-C, high density lipoprotein cholesterol; LDL-C, low density lipoprotein cholesterol; Q, quintile; TEI, total energy intake

[a] median, (range) 2.9 (2.4 - 3.5) vs 0.4 (0 - 0.8)

[b] Fully adjusted model is adjusted for age, sex, ethnicity, Townsend deprivation index, education, smoking, alcohol consumption, physical activity, dietary supplements and consumption of vegetables, spread-type, non-oily fish, oily fish, red unprocessed meat, total processed meat, cheese, wholegrains, refined grains, coffee, decaffeinated coffee and tea

[c] Log transformed variables: Beta coefficients are presented as % change rather than mean difference; Estimates are back-transformed with exponentiation.

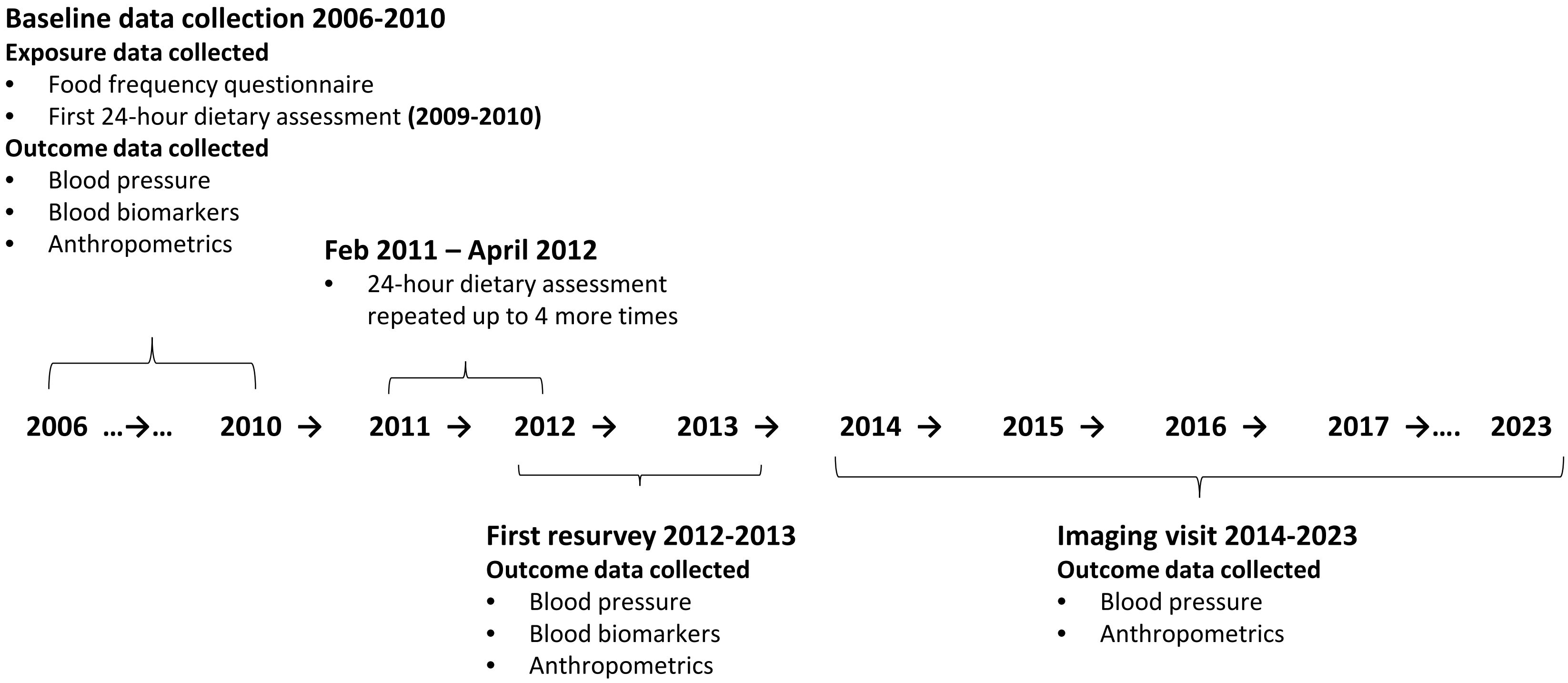

Supplemental Figure S1. Timeline of data collection in UK Biobank

Supplemental Figure S2. Adjusted means of cardiometabolic risk factors at BASELINE (2009−2010) by BASELINE mean fruit intake (servings/day) among those completing 2 to 5 WebQ 24−h dietary assessments (2009−2012)

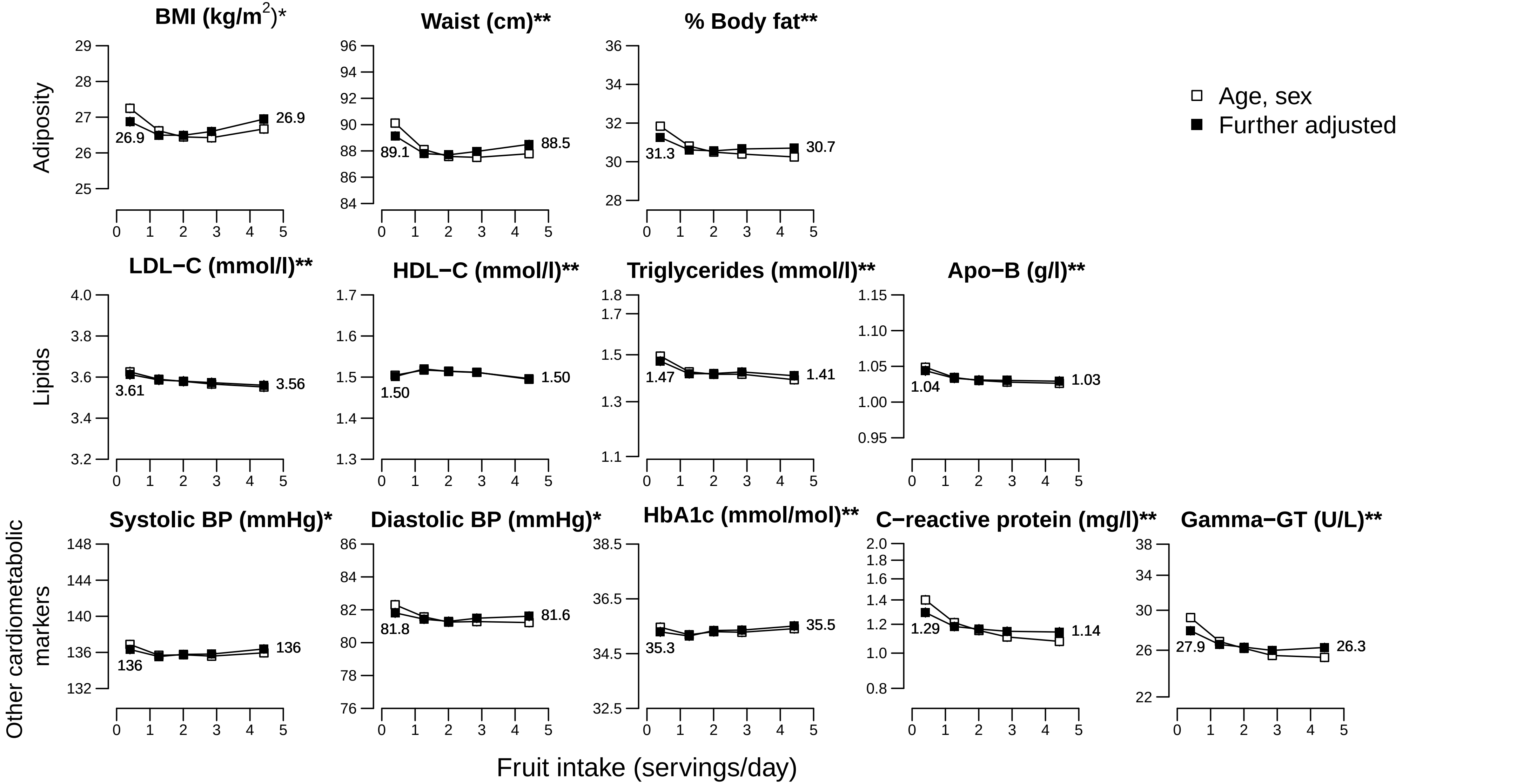

Apo, apolipoprotein; BMI, body mass index; BP, blood pressure; Gamma−GT, Gamma−glutamyl Transferase; HDL−C, high−density lipoprotein cholesterol; LDL−C, low−density lipoprotein cholesterol
n=26 596 for all outcomes apart from HbA1c with n=24 933; *FDR−adjusted P−value <0.05 for age, sex adjusted model; **FDR−adjusted P−value <0.05 for both models
Further−adjusted model was additionally adjusted for ethnicity, quintiles of the Townsend deprivation index, educational level, smoking status, alcohol consumption, physical activity, intakes of vegetables, whole grains, refined grains, non−oily fish, oily fish, red meat, processed meat, butter, cheese, coffee, decaffeinated coffee, tea, dietary supplements and total energy intake.
Estimates for log−transformed Triglycerides, C−reactive protein, and Gamma−GT were back−transformed with exponentiation.

Supplemental Figure S3. Adjusted means of cardiometabolic risk factors at FIRST FOLLOW−UP (2012−2013) by BASELEINE mean fruit intake (servings/day) among those completing 2 to 5 WebQ 24−h dietary assessments (2009−2012)

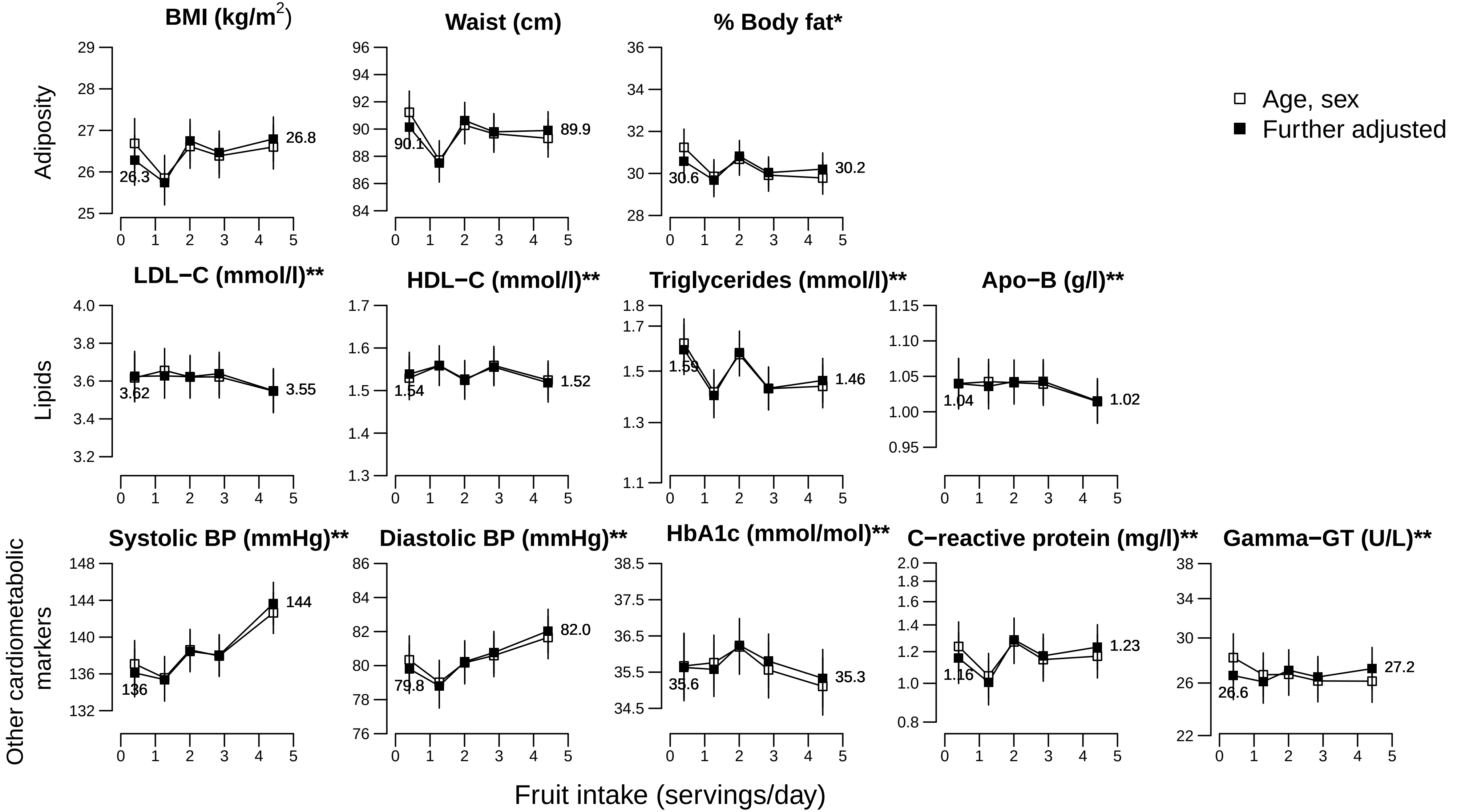

Apo, apolipoprotein; BMI, body mass index; BP, blood pressure; Gamma−GT, Gamma−glutamyl Transferase; HDL−C, high−density lipoprotein cholesterol; LDL−C, low−density lipoprotein cholesterol
n=1092 for all outcomes apart from HbA1c with n=650; *FDR−adjusted P−value <0.05 for age, sex adjusted model; **FDR−adjusted P−value <0.05 for both models
Further−adjusted model was additionally adjusted for ethnicity, quintiles of the Townsend deprivation index, educational level, smoking status, alcohol consumption, physical activity, intakes of vegetables, whole grains, refined grains, non−oily fish, oily fish, red meat, processed meat, butter, cheese, coffee, decaffeinated coffee, tea, dietary supplements and total energy intake.
Estimates for log−transformed Triglycerides, C−reactive protein, and Gamma−GT were back−transformed with exponentiation.

Supplemental Figure S4. Adjusted means of cardiometabolic risk factors at SECOND FOLLOW−UP (2014−2023) by BASELINE mean fruit intake (servings/day) among those completing 2 to 5 WebQ 24−h dietary assessments (2009−2012)

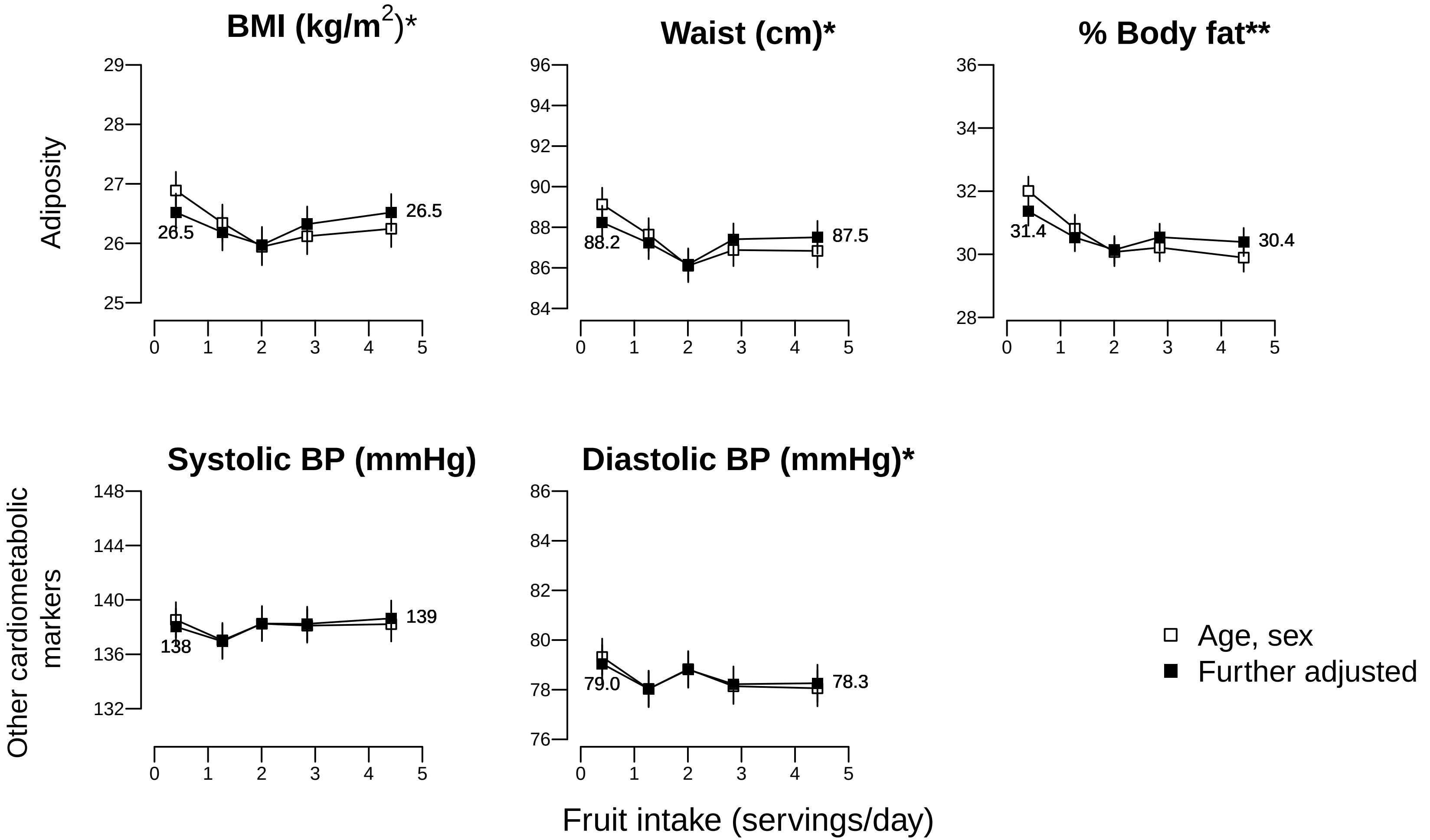

BMI, body mass index; BP, blood pressure
n=3730
*FDR−adjusted P−value <0.05 for age, sex adjusted model; **FDR−adjusted P−value <0.05 for both models
Further−adjusted model was additionally adjusted for ethnicity, quintiles of the Townsend deprivation index, educational level, smoking status, alcohol consumption, physical activity, intakes of vegetables, whole grains, refined grains, non−oily fish, oily fish, red meat, processed meat, butter, cheese, coffee, decaffeinated coffee, tea, dietary supplements and total energy intake.

Supplemental Figure S5. Adjusted means of cardiometabolic risk factors at BASELINE (2009−2010) by BASELINE mean fruit intake (servings/day) among those completing 2 to 5 WebQ 24−h dietary assessments (2009−2012), with and without restriction for assessments reporting typical diet only

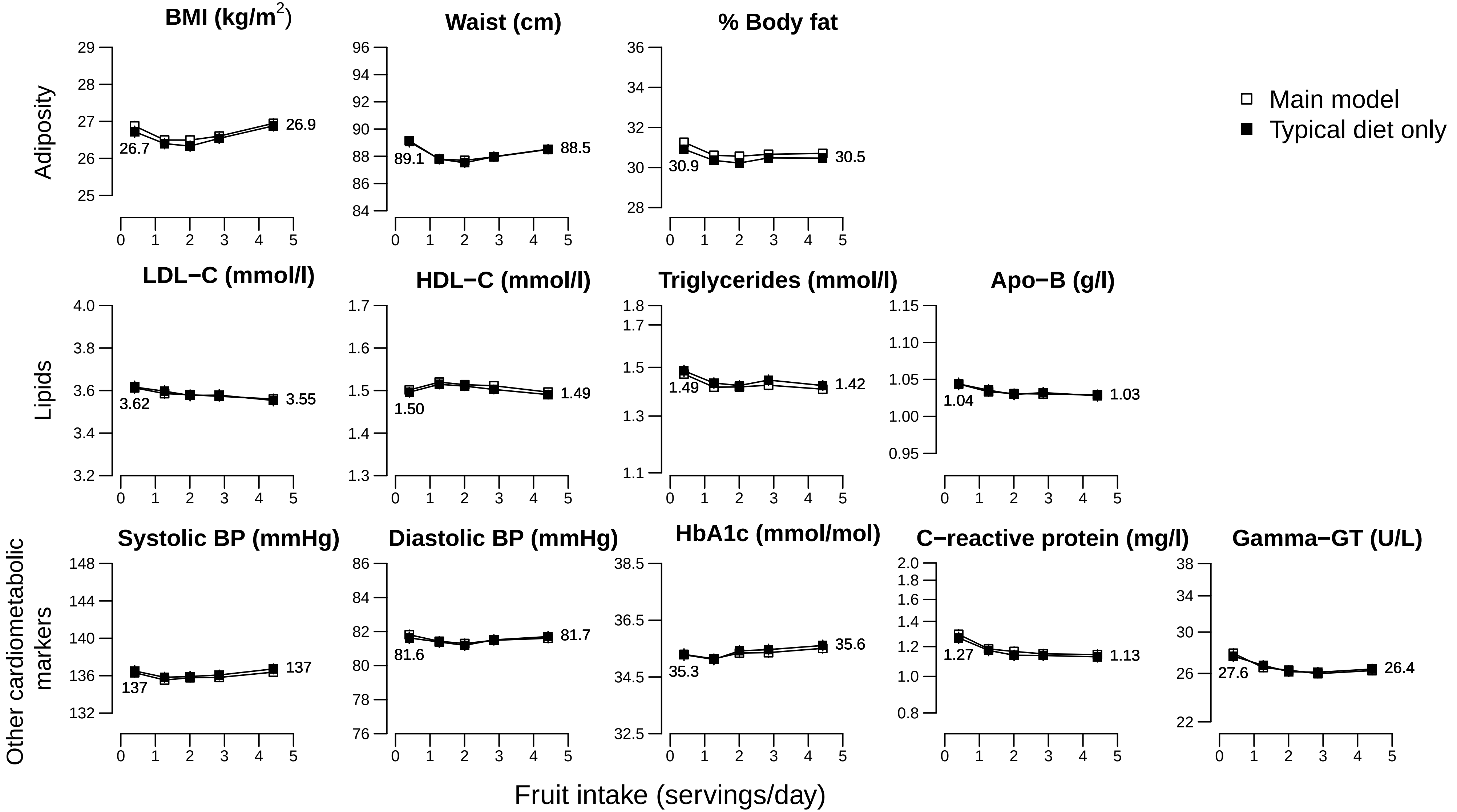

Apo, apolipoprotein; BMI, body mass index; BP, blood pressure; Gamma−GT, Gamma−glutamyl Transferase; HDL−C, high−density lipoprotein cholesterol; LDL−C, low−density lipoprotein cholesterol
Main model, n=26 596 (HbA1c n=24 933); typical diet only, n=19 702 (HbA1c=18 462)
Models were adjusted for age, sex, ethnicity, quintiles of the Townsend deprivation index, educational level, smoking status, alcohol consumption, physical activity,
intakes of vegetables, whole grains, refined grains, non−oily fish, oily fish, red meat, processed meat, butter, cheese, coffee, decaffeinated coffee, tea, dietary supplements and total energy intake.
Estimates for log−transformed Triglycerides, C−reactive protein, and Gamma−GT were back−transformed with exponentiation.

Supplemental Figure S6. Adjusted means of cardiometabolic risk factors at FIRST FOLLOW−UP (2012−2013) by BASELEINE mean fruit intake (servings/day) among those completing 2 to 5 WebQ 24−h dietary assessments (2009−2012), with and without restriction for assessments reporting typical diet only

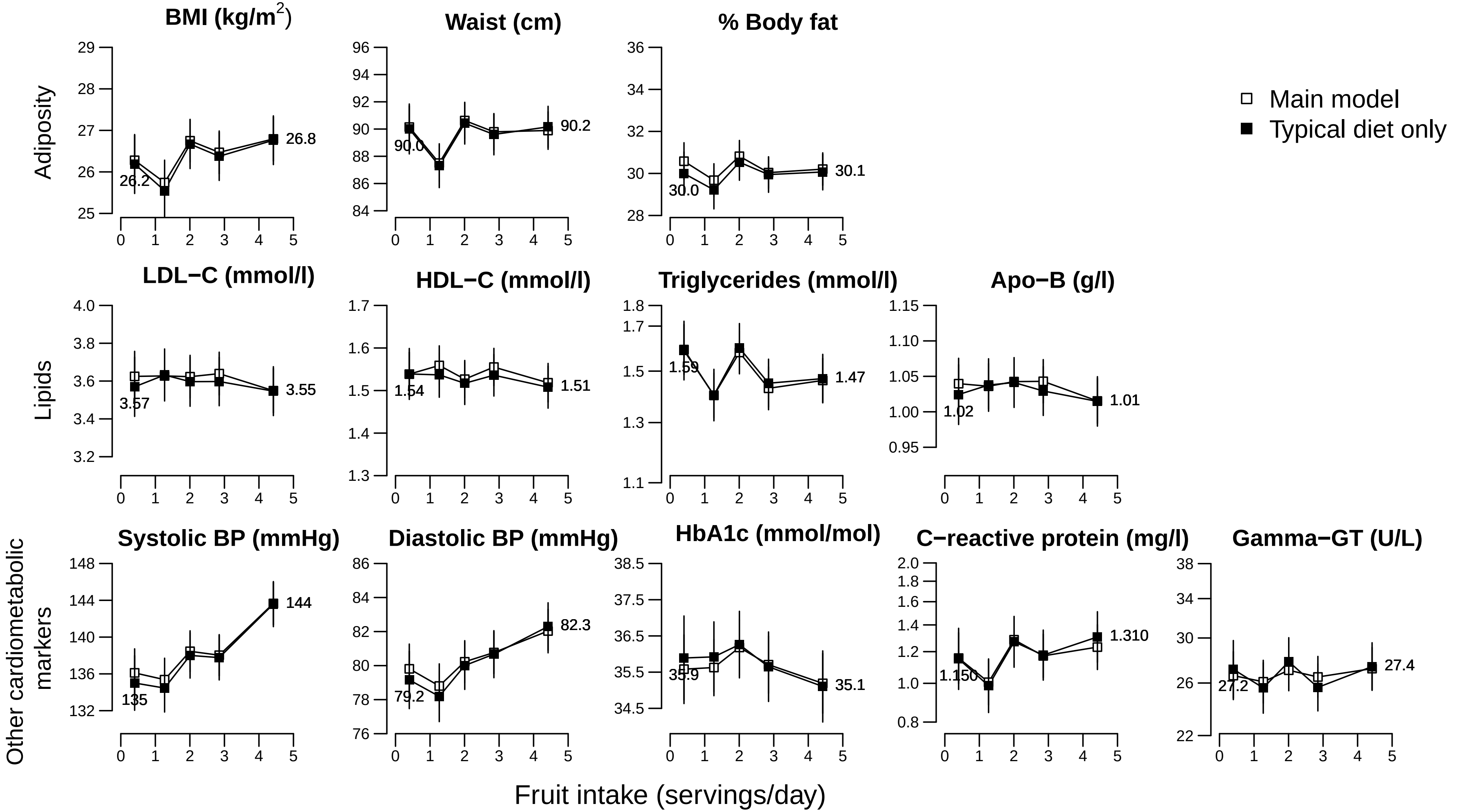

Apo, apolipoprotein; BMI, body mass index; BP, blood pressure; Gamma−GT, Gamma−glutamyl Transferase; HDL−C, high−density lipoprotein cholesterol; LDL−C, low−density lipoprotein cholesterol
Main model, n=1092 (HbA1c n=650); typical diet only, n=848 (HbA1c n=486)
Models were adjusted for age, sex, ethnicity, quintiles of the Townsend deprivation index, educational level, smoking status, alcohol consumption, physical activity, intakes of vegetables, whole grains, refined grains, non−oily fish, oily fish, red meat, processed meat, butter, cheese, coffee, decaffeinated coffee, tea, dietary supplements and total energy intake.
Estimates for log−transformed Triglycerides, C−reactive protein, and Gamma−GT were back−transformed with exponentiation.

Supplemental Figure S7. Adjusted means of cardiometabolic risk factors at SECOND FOLLOW−UP (2014−2023) by BASELINE mean fruit intake (servings/day) among those completing 2 to 5 WebQ 24−h dietary assessments (2009−2012), with and without restriction for assessments reporting typical diet only

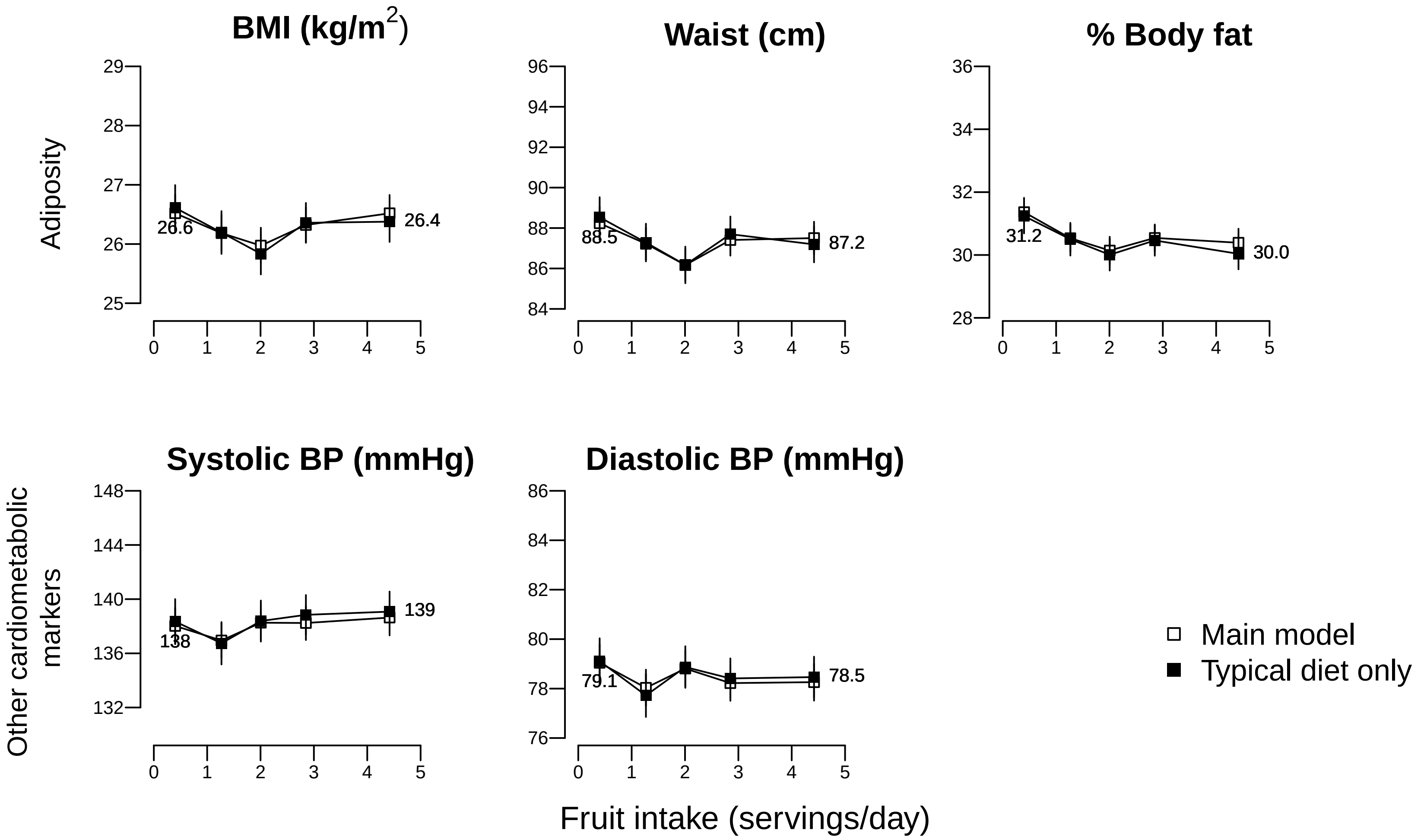

BMI, body mass index; BP, blood pressure
Main model, n=3730; typical diet only, n=2786
Models adjusted for age, sex, ethnicity, quintiles of the Townsend deprivation index, educational level, smoking status, alcohol consumption, physical activity, intakes of vegetables, whole grains, refined grains, non−oily fish, oily fish, red meat, processed meat, butter, cheese, coffee, decaffeinated coffee, tea, dietary supplements and total energy intake.
